# Molecular cloning of *AtRS4*, a seed specific multifunctional RFO synthase/galactosylhydrolase in *Arabidopsis thaliana*

**DOI:** 10.3389/fpls.2015.00789

**Published:** 2015-09-29

**Authors:** Roman Gangl, Robert Behmüller, Raimund Tenhaken

**Affiliations:** Division of Plant Physiology, Department of Cell Biology, University of SalzburgSalzburg, Austria

**Keywords:** galactinol, galactosylhydrolase, galactosyltransferase, myo-inositol, raffinose, raffinose family oligosaccharides, stachyose, stachyose synthase

## Abstract

Stachyose is among the raffinose family oligosaccharides (RFOs) one of the major water-soluble carbohydrates next to sucrose in seeds of a number of plant species. Especially in leguminous seeds, e.g. chickpea, stachyose is reported as the major component. In contrast to their ambiguous potential as essential source of carbon for germination, RFOs are indigestible for humans and can contribute to diverse abdominal disorders. In the genome of *Arabidopsis thaliana*, six putative raffinose synthase genes are reported, whereas little is known about these putative raffinose synthases and their biochemical characteristics or their contribution to the RFO physiology in *A. thaliana*. In this paper, we report on the molecular cloning, functional expression in *Escherichia coli* and purification of recombinant AtRS4 from *A. thaliana* and the biochemical characterisation of the putative stachyose synthase (AtSTS, At4g01970) as a raffinose and high affinity stachyose synthase (K_m_ for raffinose 259.2 ± 21.15 μM) as well as stachyose and galactinol specific galactosylhydrolase. A T-DNA insertional mutant in the *AtRS4* gene was isolated. Only semi-quantitative PCR from WT siliques showed a specific transcriptional *AtRS4* PCR product. Metabolite measurements in seeds of *ΔAtRS4* mutant plants revealed a total loss of stachyose in *ΔAtRS4* mutant seeds. We conclude that *AtRS4* is the only stachyose synthase in the genome of *A. thaliana* that *AtRS4* represents a key regulation mechanism in the RFO physiology of *A. thaliana* due to its multifunctional enzyme activity and that *AtRS4* is possibly the second seed specific raffinose synthase beside *AtRS5*, which is responsible for Raf accumulation under abiotic stress.

## Introduction

Seeds of higher plants are entirely dependent on their stored reserves for metabolism and growth. They contain a wide range of storage compounds, among which carbohydrates occupy a special position. Seed carbohydrates make up for the most of the carbon reserves in many seeds (Bewley and Black, 1994). α-galactosides, including raffinose family oligosaccharides (RFOs) (Han and Baik, 2006; Lahuta et al., 2010), rank next to sucrose (Suc) among water-soluble carbohydrates (WSCs) (Frias et al., 1999).

RFOs [Suc-(Gal)_n_, 1 ≤ n < 13] are water-soluble but non-reducing and non-structural derivatives of Suc to which galactosyl units are added to the glucose (Glc) moiety of Suc through α-1,6-bonds. Raffinose [Raf, Suc-(Gal)_1_] contains one galactosyl unit, whereas stachyose [Sta, Suc-(Gal)_2_] has two such galactosyl units. Raf and Sta, the most prominent members of the RFOs, get accumulated in storage organs during later stages of development (Peterbauer et al., 2001).

**FIGURE 1:**
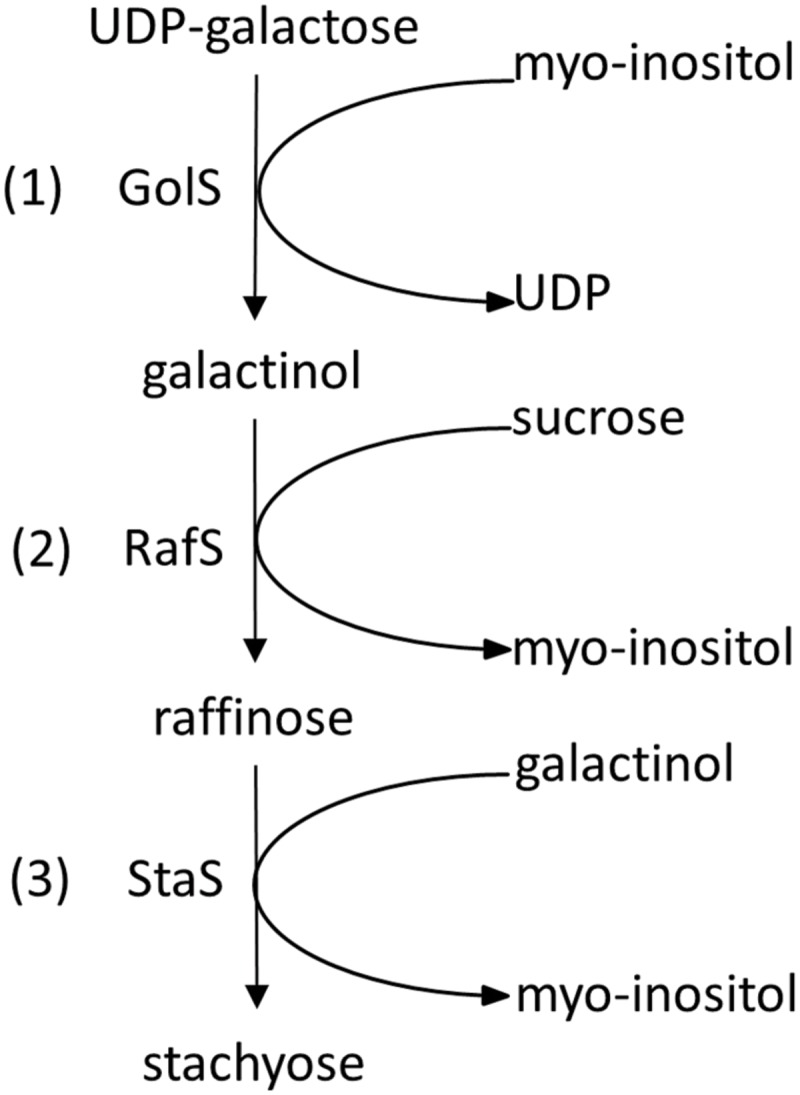
**Biosynthetic pathway of Raf and Sta in Plants.** Sta biosynthesis is initiated by galactinol synthase (GolS) (1). Biosynthesis of Sta proceeds by stepwise transfer of galactosyl units involving raffinose synthase (RafS) (2) and stachyose synthase (StaS) (3).

The first step in the RFO biosynthesis is initiated by galactinol synthase (GolS, *AtGS1-10*; EC 2.4.1.123; Pharr et al., 1981) which catalyzes the formation of galactinol (Gol), using UDP-galactose and myo-inositol (Ino) as substrates (Liu et al., 1995, 1998) (**Figure 1**, reaction 1). Biosynthesis of RFOs proceeds by stepwise transfer of galactosyl units. The second step involves raffinose synthase (RafS, *AtRS5*, EC 2.4.1.82) (Lehle et al., 1970; Lehle and Tanner, 1973) which transfers the galactosyl unit from Gol to the C6 position of the Glc unit in Suc, forming an α-1,6-galactosidic linkage to yield the trisaccharide Raf (Osumi et al., 2005, 2008; Egert et al., 2013) (**Figure 1**, reaction 2). In a third step, stachyose synthase (StaS, *AtRS4*, EC 2.4.1.67) (Tanner and Kandler, 1968; Lehle and Tanner, 1973) transfers the galactosyl moiety from Gol to the C6 position of the galactose (Gal) unit in Raf to yield the tetrasaccharide Sta (Holthaus and Schmitz, 1991; Peterbauer and Richter, 1998; Hoch et al., 1999) (**Figure 1**, reaction 3).

Seeds of dicotyledonous species preferentially accumulate Sta and higher homologs, like verbascose [Verb, Suc-(Gal)_3_] (Kuo et al., 1988). RFOs account for more than a half of total WSCs in cowpea and soybean (Martín-Cabrejas et al., 2008). Sta is reported as the major component in chickpea and lentil flours (Sosulski et al., 1982). In *Arabidopsis thaliana (A. thaliana)* seeds, both Raf and Sta accumulate (Ooms et al., 1993; Bentsink et al., 2000; Nishizawa-Yokoi et al., 2008). In *A. thaliana* vegetative tissues, the only RFO to accumulate is Raf, occurring mainly during exposure to abiotic stress (Taji et al., 2002; Nishizawa et al., 2008).

Raf as well as Sta appears during the later stages of seed development, get accumulated in storage organs and disappear upon germination (Obendorf, 1997). After imbibition and during early stage of seed germination, RFOs get mobilized by α-galactosidase (EC 3.2.1.22) and rapidly disappear (Zhao et al., 2006). Their breakdown is often completed before polymeric carbohydrates are mobilized (Vidal-Valverde et al., 2002; El-Adawy et al., 2003). Seeds have a high demand for energy during early germination. Since neither polymeric carbohydrates nor proteins or oils are able to meet this demand, as their breakdown is a matter of several days rather than of hours (Bewley and Black, 1994), it has been hypothesized, that RFOs as an essential source of rapidly metabolizable carbon for early germination events (Downie and Bewley, 2000) play a special role during early seed germination and that they are required for successful germination (Blöchl et al., 2007), but contradictory results scrutinize the exact role of RFOs during germination and seed development (Dierking and Bilyeu, 2009).

In contrast to their potential for promoting seed germination (Elsayed et al., 2013), RFOs particularly in leguminous seeds are indigestible due to lack of α-galactosidase in the digestive tract and therefore anti-nutritional oligosaccharides for monogastric animals as well as for humans when consumed. Because of their indigestible α-galactosidic linkages, RFOs end up in the large intestine, where they are fermented by the intestinal microflora and promote the growth of resident indigenous lactic acid bacteria (Liu et al., 2014). The microbial RFO breakdown produces a great deal of deleterious gasses (Cristofaro et al., 1974; Swennen et al., 2006; Kumar et al., 2010). Although there is some debate about the potential health benefits of non-digestible oligosaccharides (Delzenne and Roberfroid, 1994; Roberfroid, 2002), various physico-mechanical treatments and breeding programs have been reported to reduce RFO concentrations in seeds (Aguilera et al., 2009; Devindra et al., 2011). Even the enzymatic removal of RFOs from seed-derived products (Kulkarni et al., 2006; Patil et al., 2009) using immobilized α-galactosidase from *Aspergillus oryzae*, has been extensively studied (Slominski et al., 1994; Khalil and Mansour, 1998; Feng et al., 2008; Liu et al., 2014). To improve nutritional quality of leguminous seeds, RFO concentrations need to be reduced (Qiu et al., 2015) without affecting their role during seed development. Therefore, the key regulating steps of RFO biosynthesis need to be identified.

Stachyose synthase is an important enzyme involved in the biosynthesis of RFOs, which occur frequently in higher plants, and has been identified and partially characterized from several sources. Generally, plant StaSs are poorly biochemically characterized, because of difficulties in expression and especially purification of functional recombinant protein. Until now, the only biochemical characterisation of StaS either as purified enzyme or from crude extracts of transformed insect cell lysate has been reported from Lentil seed (Hoch et al., 1999) and Adzuki bean (Peterbauer and Richter, 1998; Peterbauer et al., 1999). StaSs have attracted little attention, probably owing to the missed promising results on cold resistance (Iftime et al., 2011).

In this paper, we report on the molecular cloning, functional expression and purification of *AtRS4* cDNA from *A. thaliana* in *Escherichia coli* and the biochemical characterization of the putative StaS (AtSTS, At4g01970) with RafS and high affinity StaS as well as Sta and Gol specific galactosylhydrolase enzyme activity of the recombinant AtRS4. Furthermore, metabolite measurements in seeds of *ΔAtRS4* mutant plants revealed a total loss of Sta in *ΔAtRS4* mutant seeds.

## Materials and Methods

### Plant Material and Growth Conditions

*Arabidopsis thaliana*, ecotype Columbia (wild type, WT) and *ΔAtRS4* mutant seeds, were cold-treated to synchronize germination and either grown in standard fertilized soil (type ED73) or on 0.5 × MS agar plates [solid Murashige Skoog phytagel medium: 0.5 × MS-salts (Duchefa, BH Haarlem, Netherlands), 0.3% (w/v) phytagel, 0.3% (w/v) Suc, KOH (pH 5.7)] or on 0.5% plant agar plates (Duchefa) in a short-day growth chamber under control growth conditions at 23°C with 8 h light with approximately 100 μE m^-2^ s^-1^ and 16 h dark period. During the dark phase, temperature was decreased to 18°C. Humidity was set to 60% during cultivation. After sampling leaf material, plants were transferred to a long-day growth chamber with 10 h light and 14 h dark period and conditions as described above. Before planting seeds on plates, seeds were surface sterilized. Samples were taken from seeds, siliques, and leaves of *A. thaliana* WT and *ΔAtRS4* mutant plants. Germination was defined as the time between sowing and protrusion of the radicle. Three biological replicates were grown from each line and one sample was picked from each biological replicate. One seed sample consisted of 20 mg seeds, one silique sample consisted of 30 siliques and one leaf sample consisted of two medium-sized leaves of plants that were put into pre-weighted reaction tubes together with one 3 mm stainless steel ball. After harvesting the plant material, sample tubes were weighted and immediately stored in liquid nitrogen. The plant material was pulverized in liquid nitrogen-cooled Teflon carriers for 2 min and 30 Hz using a ball mill (Model PM200, Retsch, Düsseldorf, Germany).

### *ΔAtRS4* Mutant Plant Analysis

The *ΔAtRS4* mutant plant in the Col-0 background was obtained from the SALK collection [At4g01970, (SALK_045237)] (Alonso et al., 2003). The *ΔAtRS4* mutant plant carries a T-DNA insert in the first exon of At4g01970. Genomic DNA (gDNA) was extracted from young leaves and the genetic identity of the plants determined by PCR technique. gDNA from *A. thaliana* leaves was extracted by the standard cetyl trimethylammonium bromide (CTAB) buffer method. Homozygous *ΔAtRS4* mutant plants were identified by PCR using two different primer pairs: *AtRS4* WT allele was amplified with the wtAtRS4_fwd and wtAtRS4_rev primer and *ΔAtRS4* mutant allele was amplified with the SALK left border and the wtAtRS4_rev primer (Supplementary Table S1).

### Extraction of WSCs and Sugar Alcohols

Liquid-liquid extraction according to Lunn et al. (2006) was applied to extract WSCs and sugar alcohols from plant material. 250 μl of quenching solution (chloroform to methanol in a 3:7 (v/v) ratio) were added to the pulverized samples. The samples were incubated at -20°C for 2 h and vigorously mixed every 30 min. WSCs and sugar alcohols were twice extracted by adding 400 μl of water to the organic layer and the aqueous layer was collected after centrifugation at 13,000 × *g* for 10 min. The aqueous phases containing WSCs and sugar alcohols were vaporized using a vacuum concentrator (Model 5301 Concentrator plus, Eppendorf, Vienna, Austria) at 45°C using the AL setting (setting 3), which is recommended for alcoholic solutions. After vaporization, all samples of each biological replicate were reconstituted in 500 μl water and diluted in a 1:5 (v/v) ratio with water (Behmüller et al., 2014; Gangl et al., 2014).

Recoveries of extraction of WSCs and sugar alcohols were determined individually for each WSC and sugar alcohol by extracting an aliquot of the respective WSC or sugar alcohol solution (10 μmol l^-1^) as described above and comparing the peak area obtained from triplicate high-performance anion-exchange chromatography with pulsed amperometric detection (HPAEC-PAD) measurements of the extracted solution with that of a standard solution having the same concentration (Supplementary Figure S1). Linearity of WSCs and sugar alcohols in the concentration range from 5 to 100 μmol l^-1^ was tested with HPAEC-PAD measurements (Supplementary Figure S2).

### cDNA Cloning of *AtRS4*

Total RNA was extracted from leaves of *A. thaliana*. Leaves were frozen in liquid nitrogen, homogenized with a ball mill, and extracted by TriReagent buffer method according to Chomczynski (1993). Residual DNA was removed by treatment with RNase-free DNase (Fermentas, St Leon-Rot, Germany). Single strand cDNA was synthesized from 1 μg of total leaf RNA using the RevertAid First Strand cDNA synthesis kit (Fermentas, Vienna, Austria). For cloning the *AtRS4* gene based on the cDNA sequence of *A. thaliana* two primers with restriction sites were designed, AtRS4_fwd with Sac and AtRS4_rev with Sma I restriction site (Supplementary Table S1). PCR was performed with Phusion High-Fidelity DNA polymerase (Thermo Scientific, Vienna, Austria) using AtRS4_fwd and AtRS4_rev primer and single-stranded cDNA as template under following conditions: initial denaturation at 98°C for 30 s, followed by 5 × (98°C for 10 s, 51°C for 30 s, 72°C for 90 s), 21 × (98°C for 10 s, 51 + 1°C for 30 s, 72°C for 90 s), 9 × (98°C for 10 s, 72°C for 30 s, 72°C for 90 s) and final elongation at 72°C for 10 min. The PCR product was restricted with Sac I and Sma I (Fast Digest; Thermo Scientific, Vienna, Austria), gel purified using GeneJet Gel Extraction Kit (Thermo Scientific, Vienna, Austria) and ligated into *E. coli* expression vector pQE30 (Qiagen, Hilden, Germany) using Rapid DNA Ligation Kit (Thermo Scientific, Vienna, Austria).

### Expression and Purification of Recombinant AtRS4

For expression of recombinant AtRS4 from *A. thaliana* in *E. coli* cells, the expression construct was cultivated in a 1 l Erlenmeyer flask containing 250 ml of liquid LB medium supplemented with 100 μg ml^-1^ of ampicillin at 37°C to an OD_600_ between 0.6 and 1.0 under vigorous shaking. The culture was cooled to 18°C and expression of recombinant AtRS4 was induced by addition of 1 mM Isopropyl-β-D-thiogalactopyranoside (IPTG). After 20 h vigorous shaking at 18°C, cells were cooled to 4°C for 30 min before harvesting. All following purification steps were carried out at 4°C.

The culture was split into three parts of 80 ml each, centrifuged and pellet was stored on -80°C. For purification of recombinant AtRS4, the pellet was thawed and resuspended in 2 ml of equilibration buffer (50 mM NaH_2_PO_4_, 300 mM NaCl, 1 mM DTT and a pH of 8.0 adjusted with NaOH). Lysozyme from chicken egg was added to a final concentration of 1 mg ml^-1^ (Roche Applied Science) and the solution was gently shaken for 30 min on ice. After incubation, the suspension was sonicated 10 times with 60% amplitude for 10 s with 10 s cooling between each burst on ice (Sonoplus, Bandelin, Berlin, Germany). The remaining insoluble residues were removed by centrifugation for 10 min at 13,000 *g*.

For purification of His-tagged recombinant AtRS4, the clarified supernatant was applied to pre-packed Protino^®^ Ni-TED 1000 columns (Macherey and Nagel, Düren, Germany) equilibrated with 2 ml equilibration buffer. The column was washed three times with 2 ml of equilibration buffer and bound recombinant protein was eluted with 1.5 ml of elution buffer (50 mM NaH_2_PO_4_, 300 mM NaCl, 1 mM DTT, and 250 mM imidazole and a pH of 8.0). Purified recombinant AtRS4 was analyzed by SDS-PAGE, respectively, by Western Blot, and immediately analyzed in HPAEC-PAD enzyme assays.

### Protein Determination

Protein concentration was determined on the NanoDrop^®^ ND-1000 Spectrophotometer (peqLab, VWR International GmbH, Erlangen, Germany) using the method of Bradford assay with bovine serum albumin as reference protein. Since, purification of recombinant AtRS4 showed additional bands on SDS-PAGE, we performed a quantification of recombinant AtRS4, using the Gel Quantification Analysis tool of ImageJ software (Schneider et al., 2012). The eluate consists of approximately 50% of recombinant AtRS4, which was used for enzyme assay calculations.

### SDS-PAGE and Western Blot Analysis

Different fractions, obtained during purification of recombinant AtRS4, were separated and analyzed for purity by SDS-PAGE using a 10% acrylamide separation gel and colloidal Coomassie blue staining. For Western blots, unstained gels were blotted onto a nitrocellulose membrane (Schleicher and Schuell Protran^TM^ BA85) for 1 h at 100 V. Non-specific binding sites were blocked by incubating the membrane for 1 h with TBST-BSA (1% w/v) at room temperature followed by two washing steps with TBST each for 15 min. For detection of His-tagged protein, a SuperSignal West HisProbe kit (Thermo Scientific, Vienna, Austria) was used. Luminescence detection was performed using an LAS 3000 mini imaging system (Fujifilm, Dusseldorf, Germany).

### StaS Enzyme Activity Assay

Detection of StaS enzyme activity was performed using HPAEC-PAD enzyme assays. StaS enzyme assays were carried out in 0.2 ml reaction tubes at a final volume of 100 μl containing 25 mM KH_2_PO_4_ (pH 7), 400 μM Raf, 100 μM Gol, and 335 ng of recombinant AtRS4. StaS enzyme assays were incubated in a PCR cycler (PCR cycler Primus 25 advanced, Peqlab, Polling, Austria) at 25°C for 60 min and reactions were stopped by heating the tubes to 95°C for 5 min. For StaS enzyme activity analysis, 10 μl of StaS enzyme assays were injected on the HPAEC-PAD system. In StaS enzyme assay, recombinant AtRS4 was assumed to catalyze the reaction Raf + Gol→Sta + Ino (**Figure 1**, reaction 3). Determination of StaS enzyme activity and biochemical data like StaS enzyme assay linearity, buffer system optimum, pH optimum, temperature optimum, and enzyme kinetics of the recombinant AtRS4 were performed by measurements of Sta product formation.

### RafS and StaS Enzyme Activity Assay

Detection of RafS and StaS enzyme activity was performed using HPAEC-PAD enzyme assays. RafS and StaS enzyme assays were carried out in 0.2 ml reaction tubes at a final volume of 100 μl containing 25 mM KH_2_PO_4_ (pH 7), 335 ng of recombinant AtRS4, and either 1 mM Gol and Suc in a range from 50 μM to 100 mM, or 1 mM Suc and Gol in a range from 50 μM to 100 mM. RafS and StaS enzyme assays were incubated in a PCR cycler at 25°C for 6 h and reactions were stopped by heating the tubes to 95°C for 5 min. For RafS and StaS enzyme activity analysis, 10 μl of RafS and StaS enzyme assays were injected on HPAEC-PAD system. In RafS and StaS enzyme assay, recombinant AtRS4 was assumed to catalyze the reaction Suc + Gol→Raf + Ino (**Figure 1**, reaction 2) and subsequently the reaction Raf + Gol→Sta + Ino (**Figure 1**, reaction 3). Determination of RafS and StaS enzyme activity and Raf to Sta product formation ratio of the recombinant AtRS4 were performed by measurements of Raf and Sta product formation.

### Gol, Raf and Sta Galactosylhydrolase Enzyme Activity Assay

Detection of galactosylhydrolase enzyme activity was performed using HPAEC-PAD enzyme assays. Galactosylhydrolase enzyme assays were carried out in 0.2 ml reaction tubes at a final volume of 100 μl containing 25 mM KH_2_PO_4_ (pH 7), 335 ng of recombinant AtRS4 and 1 mM of different substrates (either Gol, Raf, or Sta). Galactosylhydrolase enzyme assays were incubated in a PCR cycler at 25°C for 60 min and reactions were stopped by heating the tubes to 95°C for 5 min. For galactosylhydrolase enzyme analysis, 10 μl of galactosylhydrolase enzyme assays were injected on HPAEC-PAD system. In galactosylhydrolase enzyme assay, recombinant AtRS4 was assumed to catalyze the reaction Gol→Gal + Ino, reaction Raf→Suc + Gal, and reaction Sta→Raf + Gal. Determination of galactosylhydrolase activity and of enzyme kinetics of the recombinant AtRS4 were performed by measurements of Gal product formation.

### High-Performance Anion-Exchange Chromatography with Pulsed Amperometric Detection (HPAEC-PAD) of WSCs

High-performance anion-exchange chromatography separation of WSCs produced during HPAEC-PAD enzyme assays as well as WSCs extracted from plant material was performed on a HPAEC (model ICS300, Dionex Corporation, Sunnyvale, CA, USA) consisting of an ICS3000 single pump, ICS3000 electrochemical detector and a Dionex AS autosampler (Dionex Corporation, Sunnyvale, CA, USA). For chromatography of WSCs, data were analyzed with Chromeleon 7.12 (Thermo Scientific, Vienna, Austria).

For baseline separation of Gal, Suc, Raf, Sta, and Verb as well as separation of Ino and Gol a Dionex Carbopac PA20 column (150 mm × 3 mm i.d., 6.5 μm particle size) with a PA20 guard column (30 mm × 3 mm i.d., 6.5 μm particle size) was in use. Ino and Gol were separated on a Dionex Carbopac MA1 column (250 mm × 4 mm i.d., 7.5 μm particle size) with a MA1 guard column (50 mm × 4 mm i.d., 7.5 μm particle size). During measurements the column oven was set to 30°C. The mobile phase consisted of solvent A, 200 mM NaOH, and solvent B, 15 mM NaOH.

Sample measurements for the analysis of WSCs extracted from plant material, especially Raf and Sta, were performed using a Dionex Carbopac PA20 column with a gradient program that employed the starting conditions of 5% A and 95% B at a flow rate of 0.450 ml min^-1^. Starting conditions were held for 5 min. Subsequently, a linear gradient was programmed within 15 min to 25% A and 75% B. These conditions were held for 5 min. From 25 to 25.1 min, starting conditions of 5% A and 95% B were restored and kept for 9.9 min (35 min total run time). Sample measurements for the analysis of WSCs produced during HPAEC-PAD enzyme assays were performed using a Dionex Carbopac PA 20 column with a shortened gradient program (18 min total run time) (Supplementary Figure S3). Sample measurements for the analysis of WSCs extracted from plant material, especially Ino and Gol, were performed using a Dionex Carbopac MA1 column with a gradient program that employed the starting conditions of 5% A and 95% B at a flow rate of 0.400 ml min^-1^, these conditions were held for 2 min. After 2 min a linear gradient was established within 13 min to 5% A and 95% B. These conditions were held for 10 min. From 25 to 25.1 min, starting conditions of 5% A and 95% B were restored and kept for 9.9 min (35 min total run time) (Supplementary Figure S4).

### Semi-Quantitative PCR (sqPCR)

Semi-quantitative PCR was carried out in 30 μl containing 15 μl 2x PCR buffer [40 mM Tris/Cl (pH 8.4), 100 mM KCl, 6 mM MgCl_2_, 8% glycerol, 1% BSA-Solution (10 mg ml^-1^), 320 μM dNTP], 2 μl 1:20 diluted cDNA, 0.3 μl 100 μM primer fwd, 0.3 μl 100 μM primer rev, 0.2 μl 1U recombinant Taq polymerase, at a primer annealing temperature of 55°C for 35 cycles. qAtRS4_fwd and qAtRS4_rev primer (Supplementary Table S1) were designed using QuantPrime (Arvidsson et al., 2008). Target sequence was used to amplify a fragment of the corresponding cDNA.

## Results

### Sequence Analysis

**FIGURE 2:**
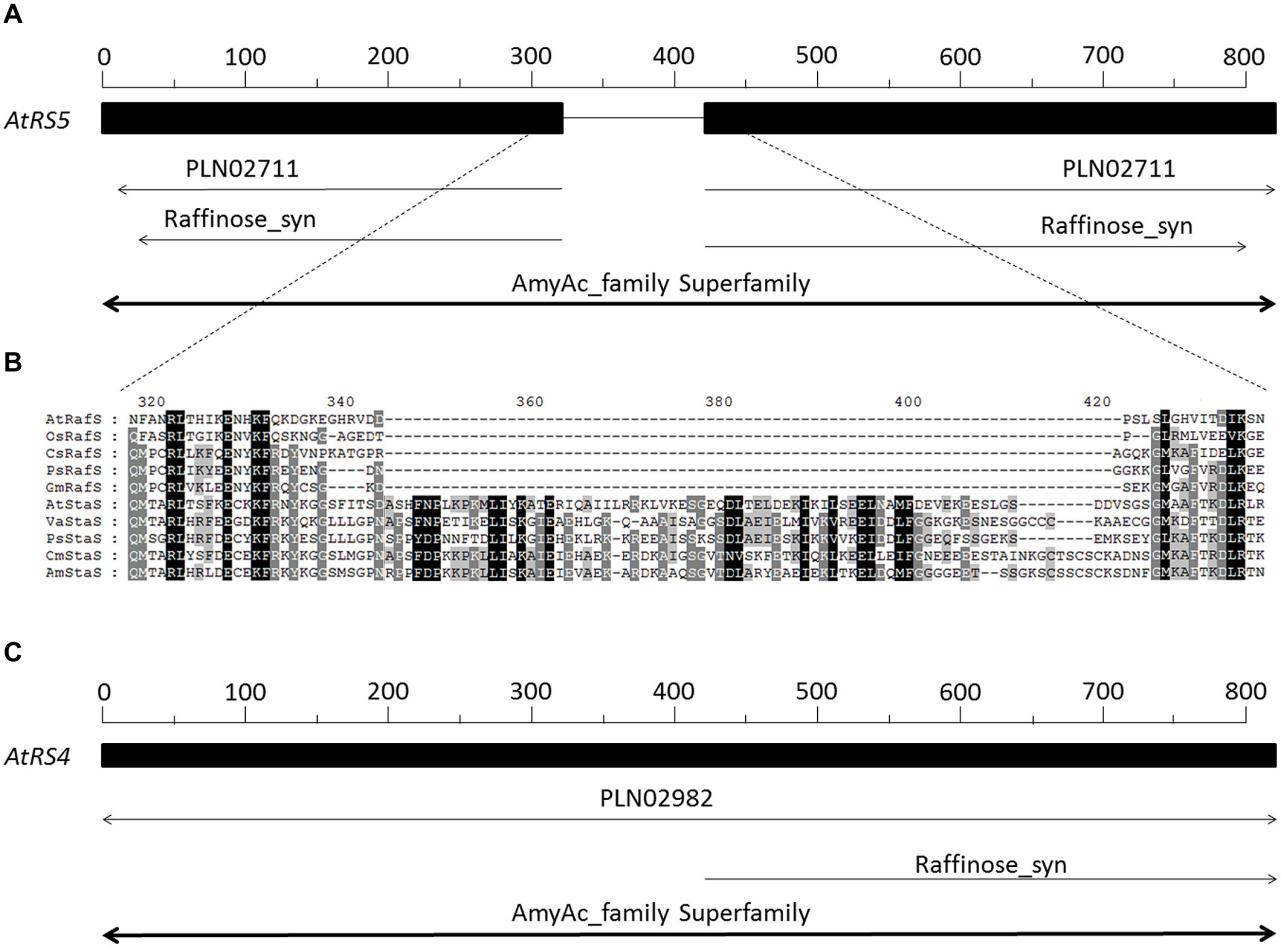
**Sequence and phylogenetic analysis of *AtRS4* and *AtRS5*. (A)** Schematically shows the 783 amino acid long sequence of *AtRS5* (A5g40390) from *Arabidopsis thaliana*. **(B)** Shows a section of a sequence alignment performed with Clustal Omega (Sievers et al., 2011) of RafS and StaS amino acid sequences, which revealed very high amino acid identity and similarity, except for a 80 amino acid long sequence block insertion, which is only present in the StaS. **(C)** Schematically shows the 876 amino acid long sequence of *AtRS4* (At4g01970) from *A. thaliana.* RafS amino acid sequence from *A. thaliana* (AtRafS, gi|332195171), *Oryza sativa* subsp *japonica* (OsRafS, gi|115471135), *Cucumis sativus* (CsRafS, gi|124057819), *Pisum sativum* (PsRafS, gi|18181865) and *Glycine max* (GmRafS, gi|187610414). StaS amino acid sequence from *A. thaliana* (AtStaS, gi|332656706), *Vigna angularis* (VaStaS, gi|6634701), *P. sativum* (PsStaS, gi|13992585), *Cucumis melo* (CmStaS, gi|659101177) and *Alonsoa meridionalis* (AmStaS, gi|21038869).

A database search using *AtRS4* sequence from *A. thaliana* clearly revealed homologous sequences in *Vigna angularis, Cucumis melo, Pisum sativum* and *Alonsoa meridionalis*, which are all coding for StaSs, as well as homologous sequences in *P. sativum, C. sativus, Glycine max*, and *Oryza sativa* subsp *japonica*, which are all coding for RafSs (**Figure 2B**). Both *AtRS4* (**Figure 2C**) as well as *AtRS5* (**Figure 2A**) amino acid sequence contain a typical motif of AmyAc_family superfamily (α-amylase catalytic domain) for α-amylase family, the largest family of glycosidehydrolases. These enzymes catalyze the transformation of α-1,4- and α-1,6-glucosidic linkages. Furthermore, the *AtRS5* (**Figure 2A**) amino acid sequence displays a typical motif of Raffinose_syn (RafS), whereas *AtRS4* only displays a 400 amino acid long C-terminal typical motif sequence (**Figure 2C**), which represents several RafSs (InterPro: IPR017853), also known as seed imbibition proteins (SIP), from the glycoside hydrolase family 36°C (InterPro: IPR008811).

It is surprising, that only a 400 amino acid long C-terminal sequence of *AtRS4* is annotated as RafS, since *AtRS4* and *AtRS5* amino acid sequence show 36% identity and 54% similarity through the whole amino acid sequence (**Table 1**). This misleading annotation is possibly based on homology differences of the N- and C- terminal sequences (**Table 1**). Despite these highly conserved amino acid sequences, one sequence block of about 80 amino acids length occur exclusively in StaS sequences (PLN02982, Gol-Raf galactosyltransferase/galactosylhydrolase) and is totally missing in RafS sequences (PLN02711, Gol-Suc galactosyltransferase) (**Figure 2B**). Thus the insertion is characteristic for StaS and allows differentiation from RafS.

**Table 1:** Identity and similarity calculation of stachyose synthase (StaS) and raffinose synthase (RafS) amino acid sequences.

Whole sequence	*VaStaS*	*PsStaS*	*CmStaS*	*AmStaS*
*AtStaS* (Identity)	56%	56%	59%	55%
*AtStaS* (Similarity)	73%	73%	75%	72%
**Whole sequence**	***OsRafS***	***CsRafS***	***PsRafS***	***GmRafS***
*AtRafS* (Identity)	56%	35%	35%	35%
*AtRafS* (Similarity)	72%	54%	52%	53%
**Whole seqence**	***AtRS1***	***AtRS2***	***AtRafS***	***AtRS6***
*AtStaS* (Identity)	29%	28%	36%	28%
*AtStaS* (Similarity)	48%	45%	54%	44%
**N-terminus**	***AtRafS***			
*AtStaS* (Identity)	28%			
*AtStaS* (Similarity)	45%			
**C-terminus**	***AtRafS***			
*AtStaS* (Identity)	35%			
*AtStaS* (Similarity)	58%			

*Calculation was performed with GeneDoc (Nicholas et al., 1999). (*AtRS1*, gi|15222768), (*AtRS2*, gi|15230330), and (*AtRS6*, gi|334187792).*

### Cloning and Expression of *AtRS4*

**FIGURE 3:**
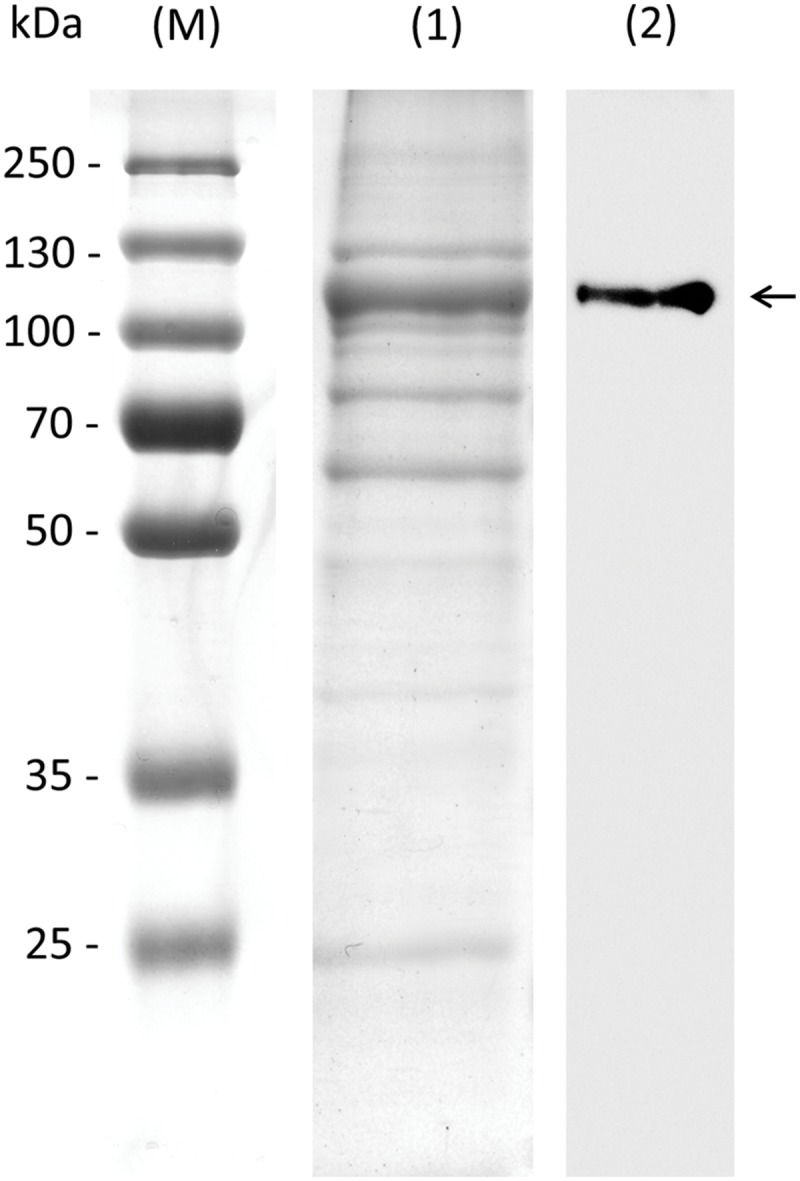
**SDS-PAGE and western blot of recombinant AtRS4.** Eluate obtained during purification of the recombinant AtRS4 was analyzed on SDS-PAGE and Western Blot. Lane (M), molecular mass marker; Lane (1), SDS-PAGE of purified recombinant AtRS4; Lane (2), Western blot of purified recombinant AtRS4.

To confirm the biochemical function of *AtRS4*, which is annotated as putative StaS with either StaS and/or RafS and/or galactosylhydrolase enzyme activity, we cloned the full-length open reading frame of *AtRS4* cDNA, encoding 876 amino acids with a calculated molecular mass of about 98.01 kDa, into the *E. coli* expression vector pQE30 with a His-tag for production of recombinant AtRS4. We obtained soluble and functional protein. SDS-PAGE of the purified soluble recombinant protein is shown in **Figure 3**. The recombinant protein was detected with a His-tag specific probe, which recognized a single band of about 100 kDa in Western blots. Expression of recombinant AtRS4 in *E. coli* culture produced 22 mg of the recombinant protein per liter of cell culture with a specific StaS enzyme activity *V*max (Raf) of 4722 pkat mg^-1^ protein.

### HPAEC-PAD Enzyme Assay Design

**FIGURE 4:**
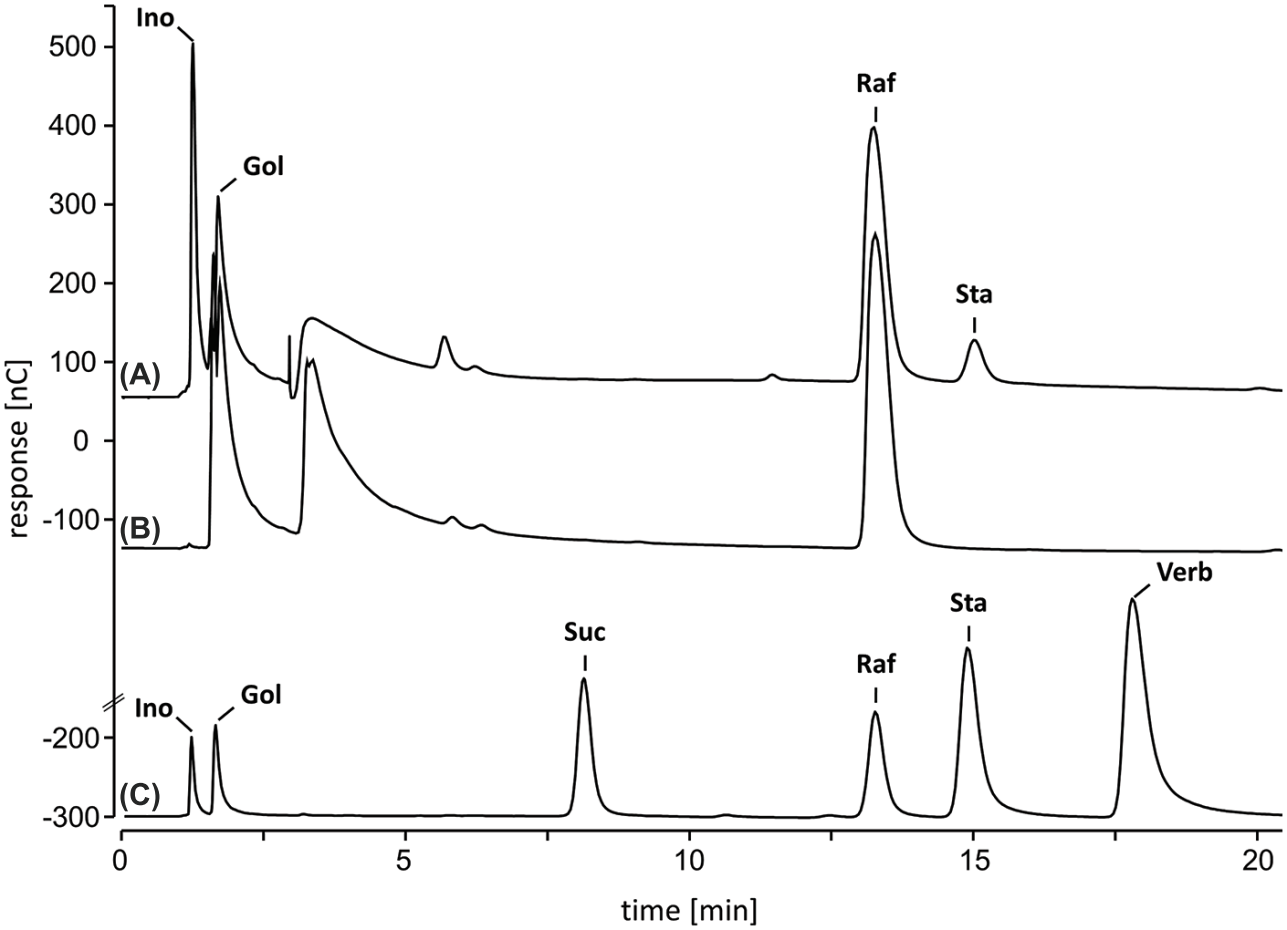
**High-performance anion-exchange chromatography with pulsed amperometric detection (HPAEC-PAD) enzyme assay.** StaS enzyme activity of recombinant AtRS4 was tested. HPAEC-PAD chromatogram **(A)** indicates Sta product formation during enzyme reaction. HPAEC-PAD chromatogram **(B)** shows control without recombinant AtRS4. HPAEC-PAD chromatogram **(C)** shows 100 μM Ino, Gol, Suc, Raf, Sta, and Verb as reference compounds.

A StaS enzyme assay was established and optimized. Furthermore, a set of stabilizing co-factors (1 mM Mg^2+^, 1 mg ml^-1^ BSA, 0.1% Triton, 0.1% Tween 20 and 1 mM DTT) was tested. To stabilize the StaS enzyme activity during the purification procedure we added 1 mM DTT to the equilibration and elution buffer for the pre-packed Protino^®^ Ni-TED 1000 columns. This was sufficient to stabilize StaS enzyme activity without further need of DTT in the StaS enzyme assay. The recombinant AtRS4 showed a detectable StaS enzyme activity on HPAEC-PAD system incubating 335 ng of recombinant AtRS4 in 25 mM K-phosphate (KOH, pH 7), 100 μM Gol and 400 μM Raf in 100 μl enzyme assays for 1 h at 25°C. Under these conditions recombinant AtRS4 catalyzes the conversion of Raf and Gol into Sta. A typical chromatogram with appropriate controls is shown in **Figure 4**. The Sta product formation is dependent on the presence of Gol and Raf and identical with the commercially available reference compound Sta. Concomitant, the intermediate Ino accumulates linearly with Sta product formation. These experiments confirm that the purified recombinant AtRS4 is indeed a StaS. Characterization of recombinant AtRS4 was performed in enzyme assays not exceeding 60 min, in which the amount of Sta increased linear with reaction time (Supplementary Figure S5). At later time points the Sta product accumulation drops, indicating that either the optimum Raf to Sta product formation ratio is reached and a galactosyl unit distribution mechanism gets activated, or that product inhibition by Ino or Sta occurs.

### StaS Characterization

**FIGURE 5:**
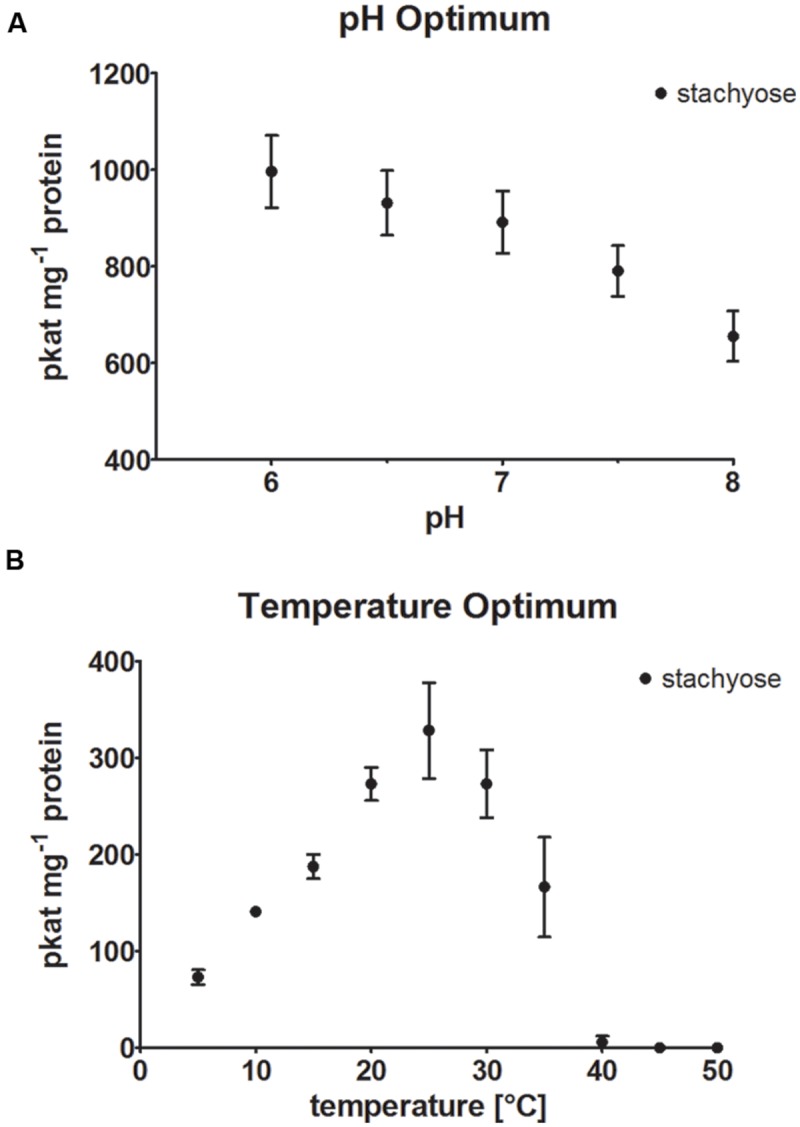
**pH and temperature optimum. (A)** The effect of different pH values on StaS enzyme activity of recombinant AtRS4 and **(B)** the dependence of StaS enzyme activity of recombinant AtRS4 on reaction temperature were measured with HPAEC-PAD enzyme assays. Sta concentration was determined using HPAEC-PAD enzyme assay. Values are averages of three independently performed assays (±SD).

StaS enzyme activity of recombinant AtRS4 was determined in different buffer systems at pH 7 and at different pH values ranging from pH 6.0 to 8.0 (**Figure 5A**), using Sta product formation as the detection method. The maximum StaS enzyme activity was reached at pH 6, whereas 65.8% of the StaS enzyme activity was measured at pH 8.0, therefore we performed enzyme assays at a neutral pH of 7 with 89.5% StaS enzyme activity. Utilization of Na-phosphate and MES buffer (pH 7), under standard conditions, showed 86.5 ± 4.8%, respectively, 82.5 ± 1.8%, of StaS enzyme activity compared with 100 ± 7.2% StaS enzyme activity in K-phosphate buffer at the same pH.

Recombinant AtRS4 showed StaS enzyme activity within a temperature range from 5 to 35°C (**Figure 5B**). The temperature optimum was located around 25°C. At 5°C still 22.2% of StaS enzyme activity was sustained, whereas 98.3% of the StaS enzyme activity was lost at 40°C caused by protein inactivation.

### Substrate Specificity and Product Formation

**FIGURE 6:**
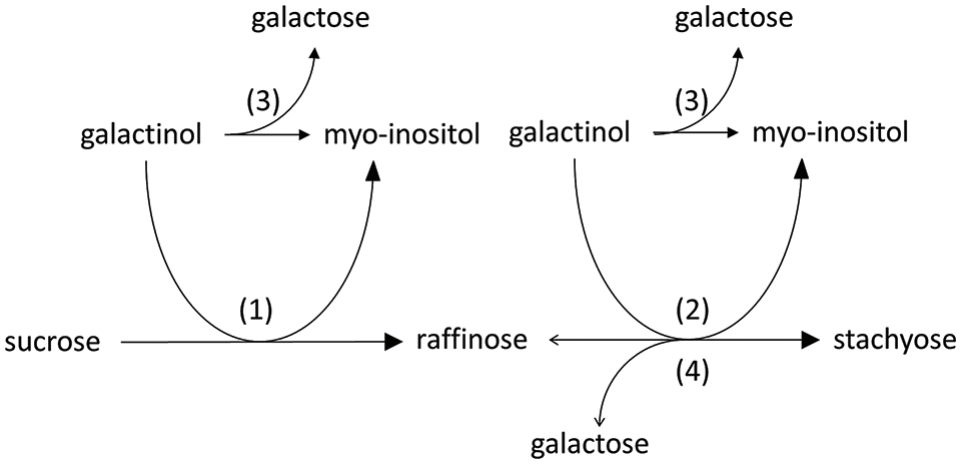
**Enzyme activities of recombinant AtRS4.** Recombinant AtRS4 annotated as putative StaS (AtSTS, AtRS4, At4g01970) shows in HPAEC-PAD enzyme assays RafS (1), high affinity StaS (2) as well as Sta (4) and Gol (3) specific galactosylhydrolase enzyme activity.

We tested the putative RafS enzyme activity, offering recombinant AtRS4 different concentration ratios of Suc and Gol for 6 h incubation at 25°C, which lead to a detectable RafS (**Figure 6**, reaction 1) as well as StaS (**Figure 6**, reaction 2) enzyme activity on HPAEC-PAD system. We could also observe the product formation of Ino and Gal in HPAEC-PAD enzyme assay controls offering recombinant AtRS4 only Gol, leading to the conclusion that recombinant AtRS4 possesses a galactosylhydrolase enzyme activity. Therefore, we tested the galactosylhydrolase enzyme activity offering recombinant AtRS4 either Sta (**Figure 6**, reaction 4) or Raf or Gol (**Figure 6**, reaction 3). Galactosylhydrolase enzyme activity could only be observed offering recombinant AtRS4 Sta or Gol. The presented results indicate that *AtRS4* has a raffinose and high affinity stachyose synthase as well as a stachyose and Gol specific galactosylhydrolase enzyme activity.

**FIGURE 7:**
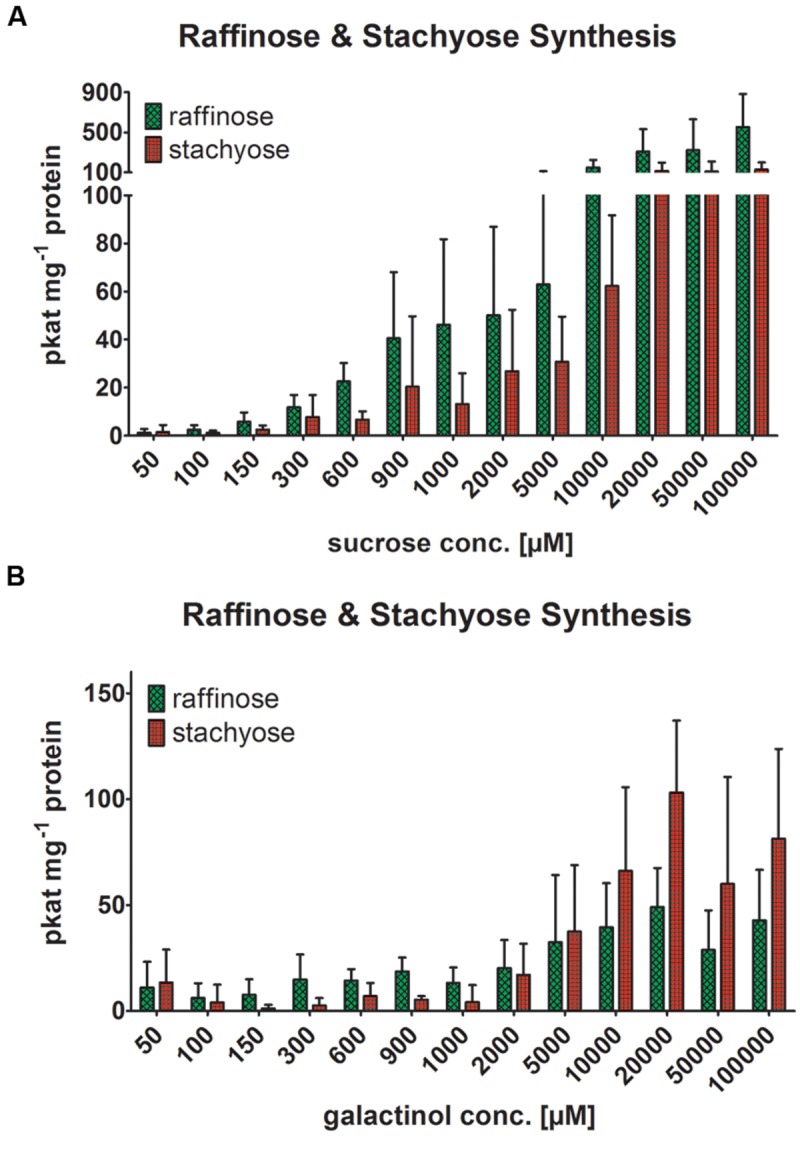
**Raf and Sta synthesis of recombinant AtRS4.** The effect of different Suc to Gol substrate concentrations ratios on the Raf to Sta product formation ratio of recombinant AtRS4 was tested. **(A)** Suc in a substrate range from 50 μM to 100 mM was incubated for 6 h with 1 mM Gol concentration and **(B)** Gol in a substrate range from 50 μM to 100 mM was incubated for 6 h with 1 mM Suc and measured with HPAEC-PAD enzyme assays. Raf and Sta concentration was determined using HPAEC-PAD enzyme assay. Values are averages of three independently performed assays (±SD).

Within 1 h recombinant AtRS4 catalyzed the conversion of Raf and Gol into Sta and Ino but failed to catalyze the conversion of Suc and Gol into Raf and Ino in HPAEC-PAD enzyme assays, indicating a very high substrate specificity of AtRS4 for Raf. Within 6 h recombinant AtRS4 catalyzed the conversion of Suc and Gol into Raf as well as Sta and Ino in HPAEC-PAD enzyme assays. Different Suc to Gol substrate concentration ratios showed impact on the Raf to Sta product formation ratio. Increasing Suc concentration with constant Gol concentration lead to an increased Raf (up to 500 pkat mg^-1^ protein) product formation (**Figure 7A**), whereas increasing Gol concentration with constant Suc concentration had no impact on Raf (approximately 100 pkat mg^-1^ protein) product formation (**Figure 7B**). Sta product formation showed in both assays similar product formation maxima, although Raf to Sta product formation ratios changed in favor of Sta offering recombinant AtRS4 higher Gol concentrations than 10 mM, due to the lack of increasing Raf product formation. Our presented results show, that *AtRS4* is a sequential multifunctional RafS and StaS as well as a high affinity StaS, accepting only Raf and Gol for Sta product formation. Additionally, our results provide evidence, that AtRS4 possesses a Sta and Gol specific galactosylhydrolase enzyme activity.

### Kinetic Analysis

**FIGURE 8:**
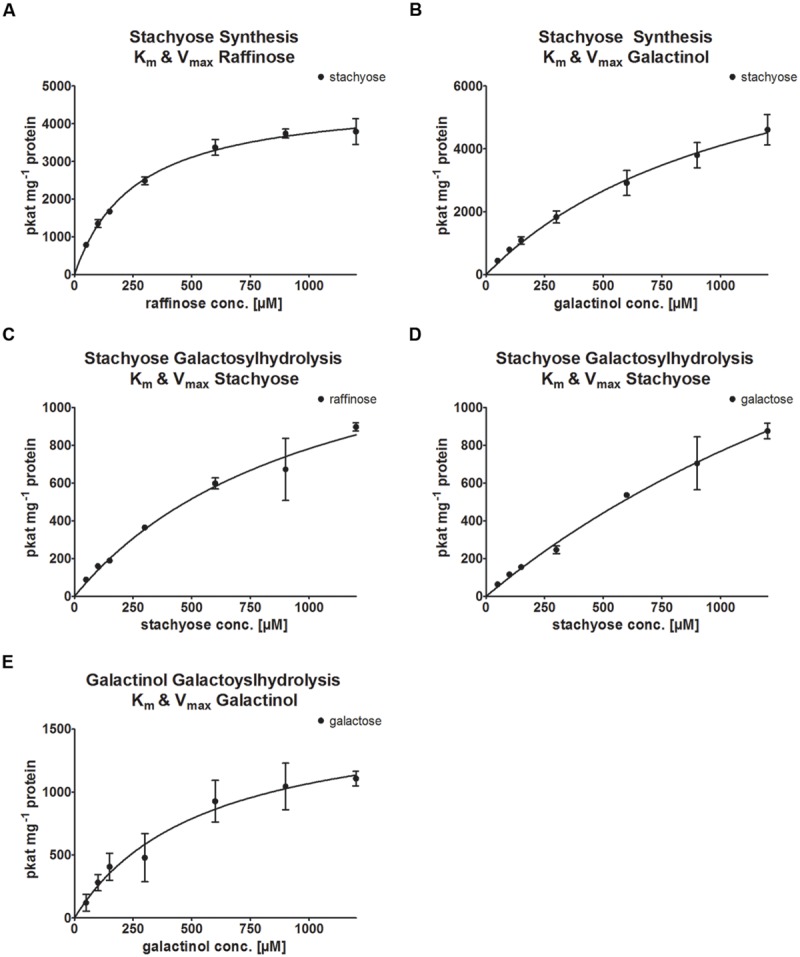
**Enzyme kinetics of recombinant AtRS4.** Enzyme activity of recombinant AtRS4 was measured with varying concentrations of substrates (50 to 1,200 μM) under standard conditions for 60 min with HPAEC-PAD enzyme assays. Values are averages of three independently performed assays (±*SD*). **(A)** Substrate saturation by Michaelis–Menten curve for Raf is shown. *K*_m_ with 259.2 ± 21.15 μM and *V*_max_ 4,722 ± 132.3 pkat mg^-1^ protein was calculated. **(B)** Substrate saturation by Michaelis–Menten curve for Gol is shown. *K*_m_ with 1,170 ± 246.8 μM and *V*_max_ with 8,911 ± 1,105 pkat mg^-1^ protein was calculated. **(C)** Substrate saturation by Michaelis–Menten curve for Sta measuring Raf is shown. *K*_m_ with 1,059 ± 269,3 μM and *V*_max_ 1,610 ± 233,3 pkat mg^-1^ protein was calculated. **(D)** Substrate saturation by Michaelis–Menten curve for Sta measuring Gal is shown. *K*_m_ with 2,832 ± 1,043 μM and *V*_max_ 2,944 ± 820.4 pkat mg^-1^ protein was calculated. **(E)** Substrate saturation by Michaelis–Menten curve for Gol measuring Gal is shown. *K*_m_ with 548.6 ± 152 μM and *V*_max_ 1,653 ± 205.3 pkat mg^-1^ protein was calculated.

Kinetic analysis of the StaS enzyme activity of recombinant AtRS4 was performed for the substrates Raf and Gol. The enzyme kinetic of recombinant AtRS4 for Raf (**Figure 8A**) showed a hyperbolic curve from which a *K*_m_ value for Raf of 259.2 ± 21.15 μM and a *V*_max_ value of 4,722 ± 132.3 pkat mg^-1^ protein was calculated using curve regression analysis with SigmaPlot 13.0 software. Substrate saturation curves of recombinant AtRS4 for Gol (**Figure 8B**) followed a hyperbolic curve according to Michaelis–Menten kinetic. The *K*_m_ value of recombinant AtRS4 for Gol in a substrate range from 50 to 1,200 μM was calculated as 1,170 ± 246.8 μM and a *V*_max_ value of 8,911 ± 1,105 pkat mg^-1^ protein.

Kinetic analysis of the galactosylhydrolase enzyme activity of recombinant AtRS4 was performed for the substrates Sta and Gol. The enzyme kinetic of recombinant AtRS4 for Sta measuring Raf (**Figure 8C**) as well as the enzyme kinetic of recombinant AtRS4 for Sta measuring Gal (**Figure 8D**) showed an intermediate curve type between linear and hyperbolic curve. When using a global fit algorithm following Michaelis–Menten kinetic a *K*_m_ value for galactosylhydrolase activity for the substrate Sta was 1,059 ± 269.3 μM and a *V*_max_ value of 1,610 ± 233.3 pkat mg^-1^ protein for product Raf and a *K*_m_ value of 2,832 ± 1043 μM and a *V*_max_ value of 2,944 ± 820.4 pkat mg^-1^ protein for the product Gal. Substrate saturation curves of recombinant AtRS4 for Gol (**Figure 8E**) followed a hyperbolic curve according to Michaelis–Menten kinetic. The *K*_m_ value of recombinant AtRS4 for Gol in a substrate range from 50 to 1,200 μM was calculated as 548.6 ± 152 μM and a *V*_max_ value of 1,653 ± 205.3 pkat mg^-1^ protein.

### Knockout Plants of *AtRS4*

**FIGURE 9:**
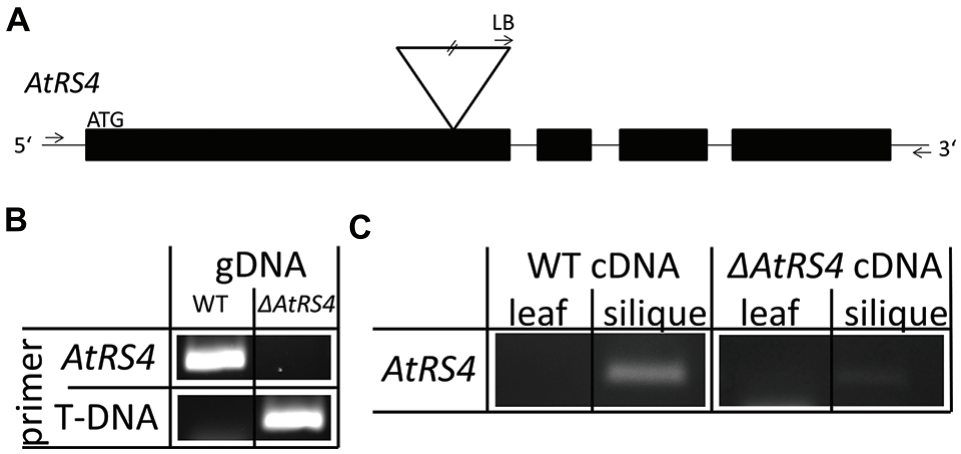
***ΔAtRS4* mutant plant analysis. (A)** Shows the localization of T-DNA insertion site in *ΔAtRS4* mutant plants. Exons are represented by black boxes and introns by lines. T-DNA is depicted by an inverted triangle. **(B)** Insertion of T-DNA was determined by PCR product obtained from gDNA of WT and *ΔAtRS4* mutant plant leaves. **(C)** Semi-quantitative PCR (sqPCR) of reverse transcribed cDNA from siliques as well as from leaves of WT and *ΔAtRS4* mutant plants.

In order to analyze the function of *AtRS4* for *A. thaliana* we isolated an insertional T-DNA mutant *ΔAtRS4.* To further characterize *ΔAtRS4* mutant plants, we isolated RNA and reverse transcribed cDNA from siliques as well as from leaves of WT and mutant plants. While sqPCR from WT siliques showed a specific transcript *AtRS4* PCR product, sqPCR from WT and *ΔAtRS4* mutant leaves and from *ΔAtRS4* mutant siliques cDNAs failed to amplify a PCR product (**Figure 9**). These results are confirmatory with expression data of At4g01970 obtained from the developmental map at the *Arabidopsis* eFP Browser (Winter et al., 2007).

### WSCs Extracts from *ΔAtRS4* Mutant Seeds Show no Detectable Sta Concentration on HPAEC-PAD

**FIGURE 10:**
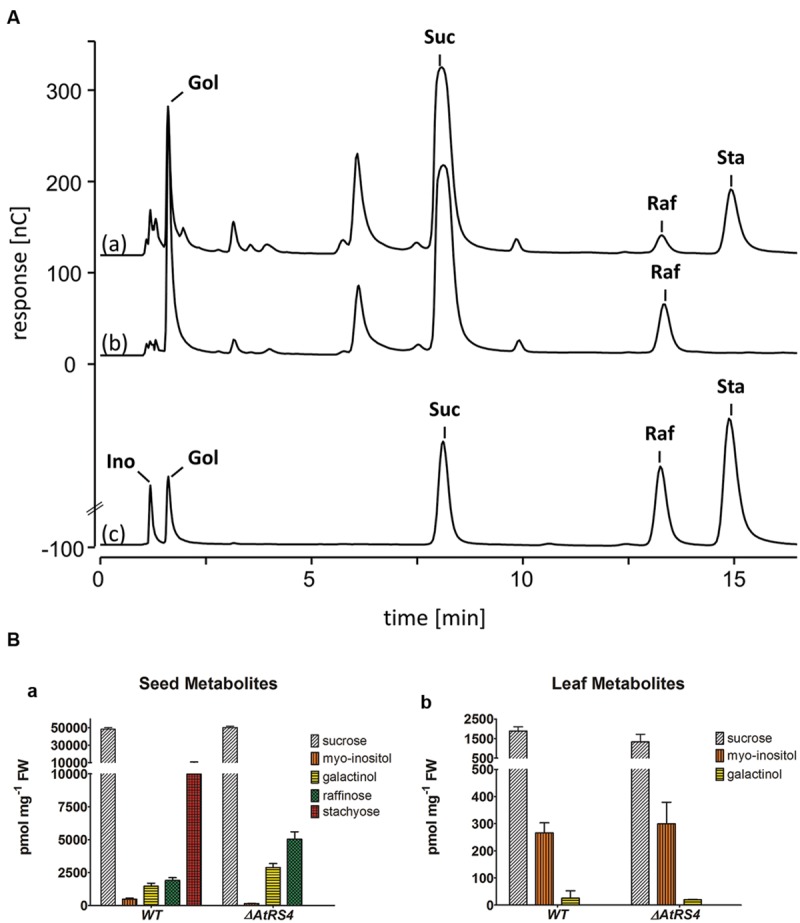
**Metabolite phenotype of *ΔAtRS4* mutant seeds. (A)** HPAEC-PAD chromatogram of water-soluble carbohydrates (WSCs) and sugar alcohols extracted from (a) WT seeds, (b) *ΔAtRS4* mutant seeds and (c) 100 μM Ino, Gol, Suc, Raf, and Sta as reference compounds. HPAEC-PAD chromatogram shows a total loss of Sta in *ΔAtRS4* mutant seeds. **(B)** Metabolite Phenotype of (a) WT and *ΔAtRS4* mutant seeds and of (b) WT and *ΔAtRS4* mutant leaves. Values are averages of three independently performed measurements (±SD).

Water-soluble carbohydrates were extracted from seeds of WT and *ΔAtRS4* mutant plants, and analyzed on HPAEC-PAD system (**Figure 10A**). The only RFOs present in WT seeds of *A. thaliana* were Raf (1,899.2 ± 216.3 pmol mg^-1^ FW) and Sta (9,976.1 ± 742.8 pmol mg^-1^ FW). The only RFO present in *ΔAtRS4* mutant plant seeds was Raf (5,040.8 ± 554.5 pmol mg^-1^ FW) and a total loss of detectable Sta on HPAEC-PAD (**Figure 10B**). In *ΔAtRS4* mutant plant seeds compared to WT plant seeds, Ino concentration was decreased by the 0.3-fold, Gol concentration was increased by the 1.9-fold and Raf concentration was increased by the 2.6-fold, occurring at concentrations of 5,040.8 ± 554.5 pmol mg^-1^ FW (**Figure 10B**).

### Seed Germination Experiments

**FIGURE 11:**
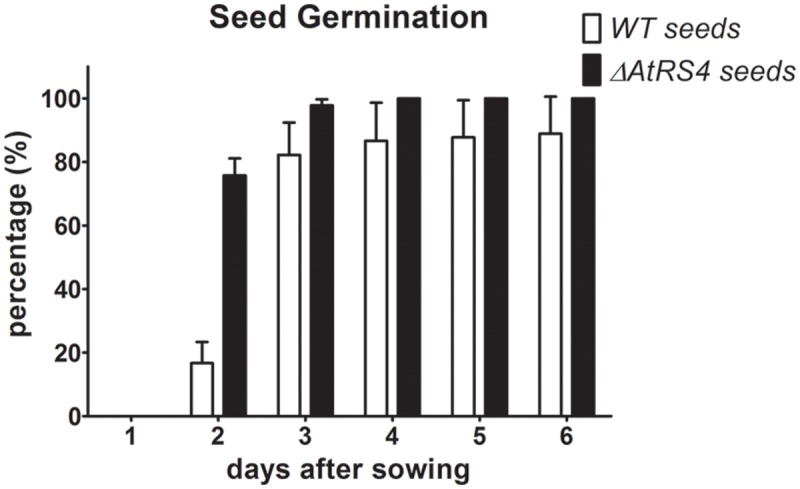
**Kinetic of WT and *ΔAtRS4* seed germination.** WT and *ΔAtRS4* mutant seeds for germination experiment were sowed on 0.5% plant agar and grew under control growth conditions. Germination was defined as the time between sowing and protrusion of the radicle. Values are averages of three independently performed experiments (±SD).

To test whether *ΔAtRS4* mutant seeds show any differences in the kinetic of imbibition and subsequent germination, we put WT and *ΔAtRS4* seeds either on 0.5 × MS agar plates or on 0.5% plant agar plates to let them germinate under standard growth conditions and observed the time period the seeds need to germinate. On 0.5 × MS agar plates, almost all *ΔAtRS4* mutant seeds started to germinate after 2 days, while WT seeds did not seem to germinate as fast as *ΔAtRS4* mutant seeds, whereas after 3 days, no difference could be observed (Supplementary Figure S6). On 0.5 × MS agar plates supplemented with 100 mM and 150 mM NaCl kinetic of germination showed no differences, while after 20 days on plates supplemented with 200 mM NaCl 66% of WT seeds and in contrast 90% of *ΔAtRS4* seeds germinated and died subsequently (Supplementary Figure S7). To avoid dormancy breaking signals like nitrate in plate media, we performed a seed germination experiment on 0.5% plant agar (Supplementary Figure S8) and observed germination of 75% of *ΔAtRS4* seeds compared to 16% of WT seeds 2 days after sowing, while 82% of WT seeds germinated 3 days after sowing (**Figure 11**). To test whether segregating seeds from a heterozygous *ΔAtRS4* mutant plant show a genotype-phenotype correlation on the kinetic of germination, we put those seeds on 0.5% plant agar plates and separated each day 5 just germinated seeds for genotyping. We did not find a correlation between the kinetic of germination and *ΔAtRS4* genotype.

## Discussion

In this study, we cloned *AtRS4* cDNA encoding a putative StaS and RafS as well as galactosylhydrolase from *A. thaliana*. The identity of the *AtRS4* cDNA was verified by functional expression in *E. coli* cells. SDS-Blot, Western-Blot and different HPAEC-PAD enzyme assays demonstrated that the *AtRS4* cDNA encodes a functional RafS and a high affinity StaS as well as a Raf and Gol specific galactosylhydrolase. The enzyme is multifunctional. The recombinant AtRS4 catalyzed the synthesis of Sta from Raf and Gol, and was able to utilize Suc and Gol as substrate to produce Raf as well as Sta. Beside the RafS and StaS enzyme activity, recombinant AtRS4 was able to hydrolyse Sta and Gol, but not Raf, providing evidence for a specific galactosylhydrolase enzyme activity of AtRS4. On 10% SDS gels, the apparent molecular mass of the recombinant AtRS4 was about 100 kDa, which is in fairly good agreement with the molecular mass of 98 kDa deduced from the amino acid sequence of the *AtRS4* cDNA.

The amino acid sequence of *AtRS4 (AtStaS)* shares high homology (36% identity and 54% similarity, **Table 1**) with *AtRS5 (AtRafS)*, an enzyme assumed to catalyze a very similar reaction. Both RafS and StaS utilize Gol as galactosyl donor and transfer them to acceptors that differ only in one galactosyl unit. However, despite these molecular and biochemical similarities, identified StaSs are totally inactive on Suc (Peterbauer and Richter, 1998; Hoch et al., 1999), while RafSs are inactive on Raf as acceptor (Lehle and Tanner, 1973). In this study, we could carve bioinformatically as well as biochemically out, that a single 80 amino acid long sequence block (**Figure 2B**) clearly divides the functional sequences of RafS and StaS in plant kingdom and serves as a reliable signature for RafSs and StaSs.

Biochemical data of plant RafSs are insufficiently reported, due to obvious expression problems in *E. coli*. Putative gene sequences have been reported for few plants, including *A. thaliana*. In the genome of *A. thaliana*, six putative RafS genes (*AtRS1-6*) are annotated. Little is known about these putative RafSs and their biochemical characteristics (Peters et al., 2010) and their contribution to the RFO physiology in *A. thaliana* (Egert et al., 2013). To our knowledge in this study we could for the first time purify a functional recombinant RafS from *A. thaliana* out of *E. coli* cells and characterize it biochemically. To elucidate the biochemical functions and their contribution to the RFO physiology in *A. thaliana* the remaining putative RafS genes need to be cloned and functional expressed as recombinant protein.

Fructosyltransferases, which catalyze the synthesis of fructans from Suc, are related to invertases and are thought to have evolved from the latter by small mutational changes (Sprenger et al., 1995; Hellwege et al., 1997; Van Der Meer et al., 1998). In consideration of the high sequence homology of galactosyltransferases, including RafS and StaS, belonging to the glycoside hydrolase family 36°C, a member of the α-amylase family, this history of origins is also very likely for galactosyltransferases. Peterbauer et al. (1999) raised concerns over this theory, because no homologies between the amino acid sequence of StaS and those of α-galactosidases were found and only acidic α-galactosidases with a broad substrate specificity have been cloned (Overbeeke et al., 1989; Zhu and Goldstein, 1994; Davis et al., 1996, 1997). Distinct α-galactosidases with high specificity toward RFOs and alkaline pH optima have been identified in leaves of *Cucurbitaceae* (Gaudreault and Webb, 1983; Gao and Schaffer, 1999), but the amino acid sequences of the latter α-galactosidases had not been reported. Peters et al. (2010) could functionally identify *AtRS2* (AtSIP2, At3g575209) as an alkaline α-galactosidase with substrate specificity for Raf, but no RafS activity. Sequence research, clearly revealed also because of the high sequence homology between *AtRS2* and *AtRS4* (28% identity and 45% similarity, **Table 1**), that galactosyltransferases and alkaline α-galactosidases definitely belong to one glycoside hydrolase family, although their individual enzymatic function is not that clear and easy to predict based on bioinformatic homology searches. RafS and StaS are related to a family of unknown function called SIPs. Peterbauer et al. (2002) expressed curiosity to see whether these SIPs are glycosyltransferases or hydrolases. In this study, we could functionally identify *AtRS4* as a RafS and a high affinity StaS, respectively, as a specific galactosyltransferase, as well as a Sta and Gol specific galactosylhydrolase, indicating that a separation between galactosyltransferase and galactosylhydrolases based on amino acid sequence is very difficult.

It is well reported that mature *A. thaliana* seeds accumulate substantial quantities of Sta and Raf (Ooms et al., 1993; Bentsink et al., 2000; Nishizawa-Yokoi et al., 2008). We hypothesized that if *AtRS4* was the sole StaS in *A. thaliana* as suggested by the putative annotation in TAIR data base and no other of the five *AtRS* genes possesses StaS enzyme activity as described in the multifunctional StaS of Lentil seed (Hoch et al., 1999) and PsStaS (Peterbauer et al., 2002), then mature seeds from *ΔAtRS4* mutant plants should be devoid of Sta accumulation, since no putative galactan:galactan galactosyltransferase (GGT) (Bachmann and Keller, 1995; Haab and Keller, 2002) is annotated in the genome of *A. thaliana*. Seeds of *ΔAtRS4* mutant plants showed an absolute loss of detectable Sta on HPAEC-PAD. The concentration of Raf was increased by the 2.6-fold, whereas the concentrations of the other major seed WSCs, Ino, and Gol, were comparable between the mutants and those of WT plants (**Figure 10**). The absolute loss of Sta and the increased concentration of Raf by the 2.6-fold in *ΔAtRS4* mutant seeds confirmed our hypothesis that *AtRS4* is the sole StaS in *A. thaliana*.

Egert et al. (2013) hypothesized the existence of at least one other seed specific, as yet unidentified, RafS in *A. thaliana*, which is responsible for the partial Raf accumulation during seed development in *ΔAtRS5* mutant plants. Our biochemical characterization of recombinant AtRS4 showed RafS and StaS enzyme activity, and was able to hydrolyse Sta into Raf, which represents the reverse reaction of the StaS enzyme activity. Recombinant AtRS4 was able to convert Suc and Gol into Raf and Sta, but was not able to hydrolyse Raf into Suc. Therefore we hypothesized that if *AtRS4* would be the second seed specific *A. thaliana* RafS as suggested previously, then mature seeds from *ΔAtRS4* mutant plants would have shown complete ablation of Sta accumulation and decreased concentrations of Raf by a 0.5-fold. However, seeds of *ΔAtRS4* mutant plants showed concentrations of Raf increased by 2.6-fold, suggesting that the knockout of the multifunctional RFO synthase in the seeds of *ΔAtRS4* mutant plants lead to a tailback of Raf and therefore the expected 0.5-fold Raf decreased metabolic phenotype was covert. Our bioinformatical and biochemical results suggest *AtRS4* as the second seed specific RafS as postulated by Egert et al. (2013), whereas absolute certainty could only be given by seed metabolite measurements of a crossed double knockout *ΔAtRS4&5* plant and biochemical characterisation of *AtRS5*.

The key regulation step of RFO biosynthesis is still controversial. Some reports favor GolS as the key enzyme whereas others consider substrate accumulation of initial substrates like Ino and Suc together with other feed-back loops as regulating step (Peterbauer et al., 2001; Karner et al., 2004; Lahuta et al., 2005; Bock et al., 2009). In this study we could clearly show that the concentration of Suc, respectively, the Suc to Gol substrate concentration ratio, has an impact on the Raf to Sta product formation ratio of recombinant AtRS4. High concentrations of Suc lead to higher concentrations of Raf, whereas the Sta concentration seems Suc and Gol concentration independent. These results lead to the conclusion, that Raf accumulation is controlled by the Suc concentration. Raf product formation takes recombinant AtRS4 much longer than Sta product formation. Once recombinant AtRS4 produced Raf, degradation to Suc is blocked due to the not detectable Raf specific galactosylhydrolase enzyme activity, whereas a multifunctional enzymatic distribution of the Raf and Sta ratio needed from the plant cell to adopt to the environment or, respectively, stress condition is possible. Egert et al. (2013) stated that the Sta accumulation in WT seeds (1.24 ± 0.076 mg g^-1^ DW) and in seeds of two different *ΔAtRS5* mutant lines (1.11 ± 0.11 and 1.24 ± 0.04 mg g^-1^ DW, respectively) show equal Sta concentrations, whereas Gol (0.52 ± 0.051 and 0.54 ± 0.026 mg g^-1^ DW, respectively) increased almost threefold in seeds from the *ΔAtRS5* mutant plants. In this paper, we could show that a total loss of Sta in *ΔAtRS4* mutant seeds, leads to an increased Raf concentration by the 2.6-fold and Gol concentration by the 1.9-fold, favoring *AtRS4* as the only StaS in *A. thaliana*, and as the key regulation step of Sta biosynthesis.

Sta is thought to be an important source of energy during seed germination. It has been hypothesized, that RFOs play a special role during early seed germination and that they are required for successful germination (Blöchl et al., 2007). Based on this knowledge, we hypothesized, that we could observe a germination phenotype of *ΔAtRS4* mutant seeds, containing an increased Raf concentration by the 2.6-fold and a total loss of Sta. But contrary to our expectations of a delayed germination, we observed that, *ΔAtRS4* mutant seeds germinated slightly earlier than WT seeds. Suggesting that a loss of Sta in *A. thaliana* seeds has no impact on germination time period or that the increased Raf concentration compensates or even speeds up germination. Clear evidence of the impact of Raf and Sta on germination kinetics could be derived from future germination experiments with WT, *ΔAtRS4, ΔAtRS5*, and especially *ΔAtRS4&5* mutant seeds, since seedlings from segregating heterozygous *ΔAtRS4* plant showed no clear genotype-phenotype correlation.

Since *AtRS5* is characterized as an abiotic stress-induced RafS, *AtRS4* can be seen as a basic Raf and Sta supply multifunctional RFO synthase, which supplies the plant seed with constant Sta accumulation and its RafS activity, respectively accumulation, is controlled by the Suc concentration of the plant cell. Therefore we hypothesize, the better the plant is doing in the environment, the more Suc is produced and the more Raf is accumulated in seeds of the new generation, which provide more energy for germination and better adaption to the environmental conditions.

AtRS4 kinetics displayed much higher affinity toward Raf when assayed with Gol (*K*_m_ 259.2 μM), compared with estimated *K*_m_ values of *CmStaS* (*K*_m_ between 3.7 and 15 mM; Huber et al., 1990; Holthaus and Schmitz, 1991), *LcStaS* (*K*_m_ 9.7 mM; Hoch et al., 1999), *PsStaS* (*K*_m_ 21.1 mM; Peterbauer et al., 2002), and *VaStaS* (K_m_ 38.6 mM; Peterbauer and Richter, 1998). Those high deviations within different *K*_m_ values are likely due to the fact, that all reported StaS kinetics were performed with seed purified enzyme or transformed cell lysate with unpurified recombinant StaS. The affinity toward Gol was also higher when assayed with Raf (*K*_m_ 1170 μM), compared with estimated *K*_m_ values of *LcStaS* (*K*_m_ 5.3 mM; Hoch et al., 1999) *PsStaS* (*K*_m_ 13.9; Peterbauer et al., 2002), and *VaStaS* (*K*_m_ 15.8 mM; Peterbauer and Richter, 1998). Our performed enzyme kinetics with recombinant purified AtRS4 confirmed very high substrate specificity and the advantage of using recombinant purified enzymes.

AtRS4 specific StaS activity [*V*_max_ (Raf) 4,722 pkat mg^-1^ protein and *V*_max_ (Gol) 8,911 pkat mg^-1^ protein] was lower, but still comparable with specific StaS activity of *LcStaS* [*V*_max_ (Raf) 9.09 nkat mg^-1^ protein and *V*_max_ (Gol) 21.6 nkat mg^-1^ protein] (Hoch et al., 1999), *PsStaS* (*V*_max_ 33.7 nkat mg^-1^) and *VaStaS* [*V*_max_ (Raf) 11.7 nkat mg^-1^ protein (Peterbauer and Richter, 1998) and *V*_max_ (Raf) 0.8 nkat mg^-1^ protein (Peterbauer et al., 1999)]. Gol specific [*V*_max_ (Gal) 1653 pkat mg^-1^ protein] galactosylhydrolase activity of AtRS4 was lower than specific StaS activity, favoring the synthesis rather than the galactosylhydrolase reaction. AtRS2 kinetics displayed comparable galactosylhydrolase activity (*V*_max_ 1.80 nkat mg^-1^) with AtRS4 kinetics, whereas substrate affinity of AtRS4 was much higher toward Gol [K_m_ (Gal) 548.6 μM], compared with *K*_m_ value of AtRS2 (*K*_m_ 105 mM).

In this paper, we report on the molecular cloning, functional expression in *E. coli* and biochemical characterisation of *AtRS4* from *A. thaliana* with the help of a *ΔAtRS4* mutant plant. The purified recombinant AtRS4 protein possesses a raffinose and high affinity stachyose synthase as well as stachyose and Gol specific galactosylhydrolase activity. A total loss of stachyose in *ΔAtRS4* mutant seeds suggests that *AtRS4* is responsible for stachyose accumulation in seeds of *A. thaliana. AtRS4* represents a key regulation mechanism in the RFO physiology of *A. thaliana* due to its multifunctional enzyme activity.

## Conflict of Interest Statement

The authors declare that the research was conducted in the absence of any commercial or financial relationships that could be construed as a potential conflict of interest.
