# Supplementary material for: Molecular cloning of AtRS4, a seed specific multifunctional RFO synthase/galactosylhydrolase in Arabidopsis thaliana
Source: Front Plant Sci. 2015 Sep 29;6:789. doi: 10.3389/fpls.2015.00789 (PMC4587089; doi:10.3389/fpls.2015.00789)
Supplement: Supplementary file 1 [file Image_1.PDF]

Supplementary Material

Molecular cloning of *AtRS4*, a seed specific multifunctional RFO synthase/ galactosylhydrolase in *Arabidopsis thaliana*

Roman Gangl, Robert Behmüller and Raimund Tenhaken*

Division of Plant Physiology, Department of Cell Biology, University of Salzburg, Salzburg, Austria

*** Correspondence:**

Raimund Tenhaken

Division of Plant Physiology

Department of Cell Biology

University of Salzburg

Hellbrunnerstrasse 34,

5020 Salzburg, Austria

raimund.tenhaken@sbg.ac.at

1 Supplementary Figures and Tables

1.1 Supplementary Figures

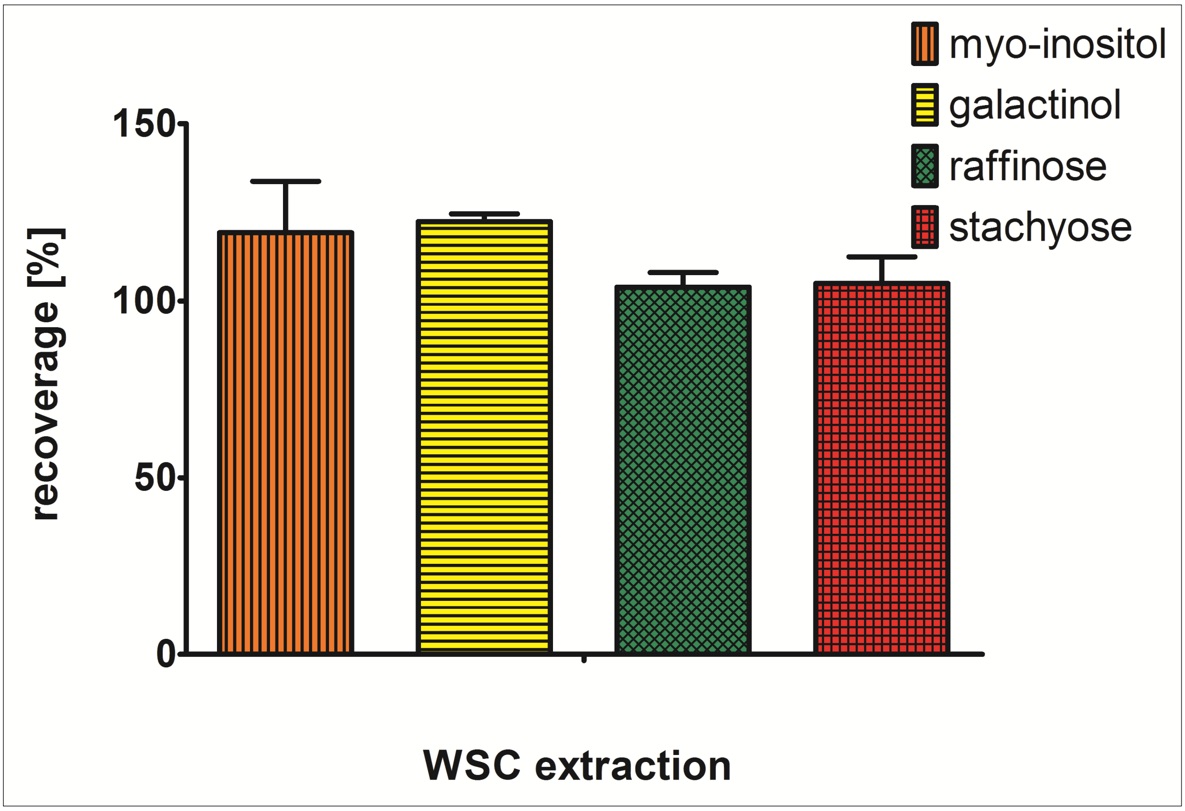

Supplementary Figure S1 Recoverage of WSCs and sugar alcohols from extraction method.

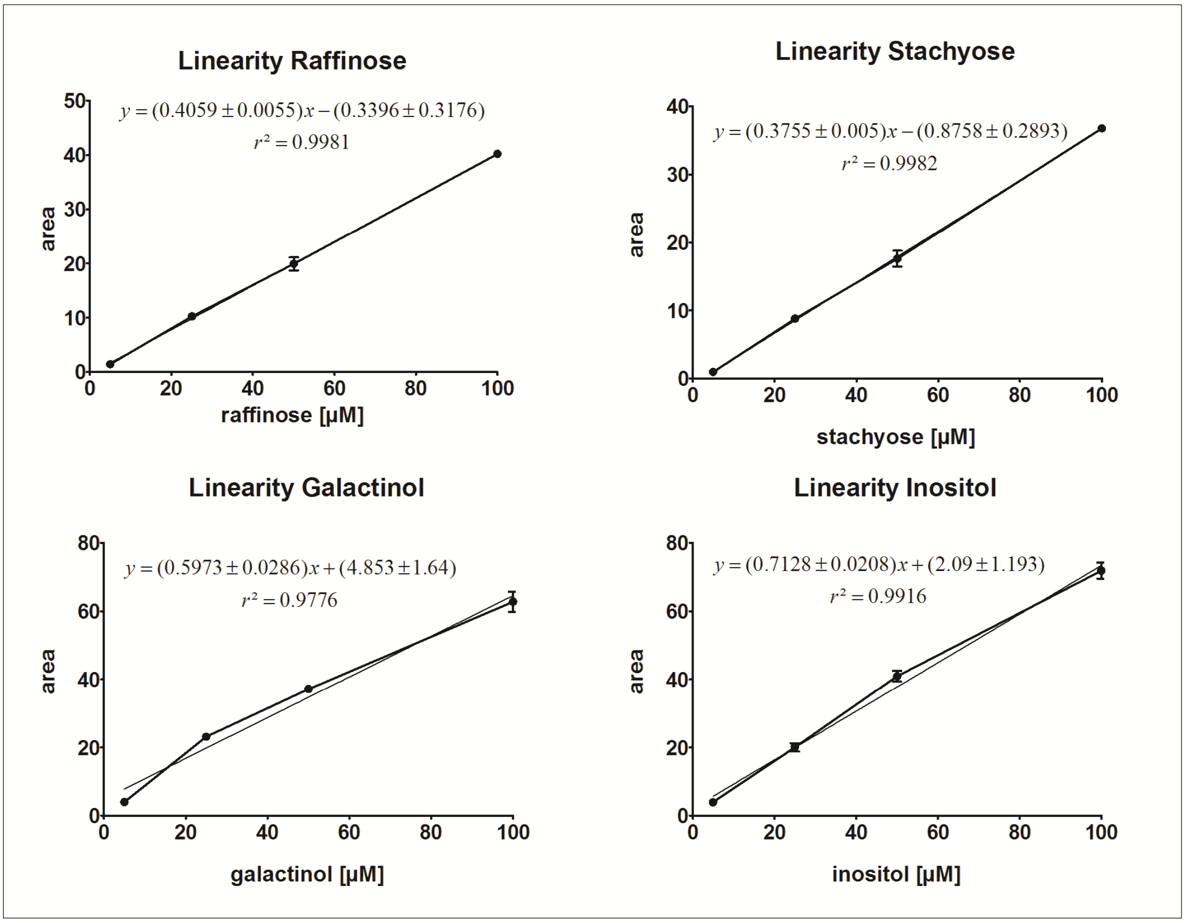

Supplementary Figure S2 Linearity of WSCs and sugar alcohols in HPAEC-PAD measurements.

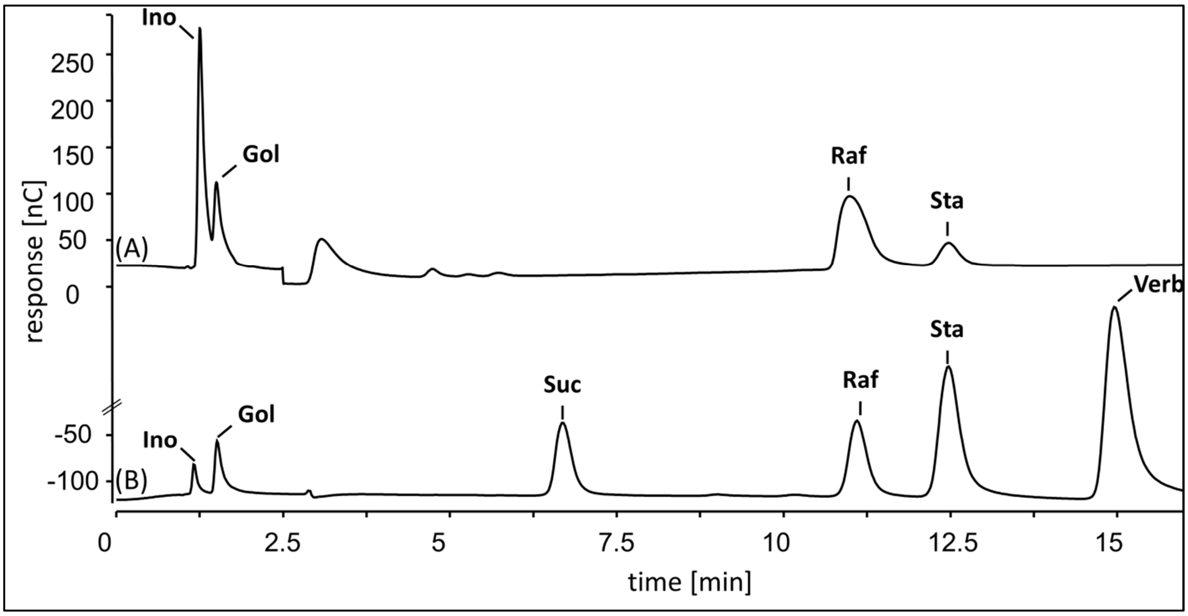

Supplementary Figure S3 HPAEC-PAD enzyme assay with shortened gradient program. StaS enzyme activity of recombinant AtRS4 was tested. HPAEC-PAD chromatogram (A) indicates Sta product formation during StaS enzyme reaction and HPAEC-PAD chromatogram (B) shows 100 µM Ino, Gol, Suc, Raf, Sta and Verb as reference compounds.

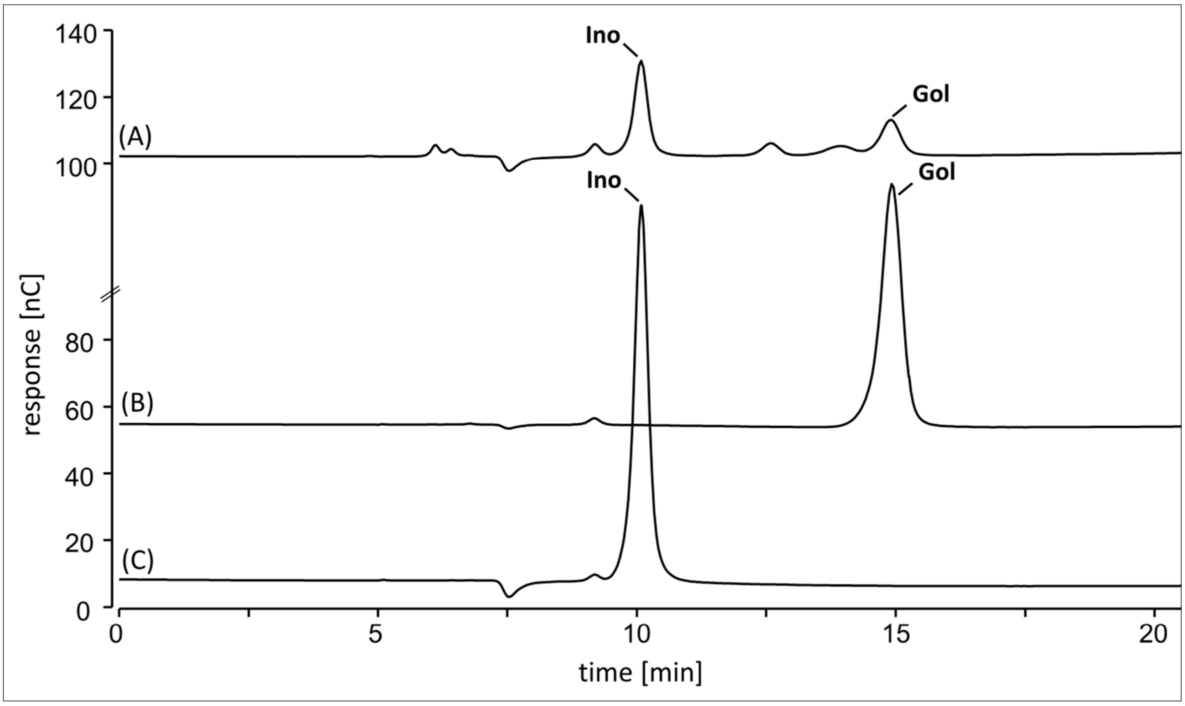

Supplementary Figure S4 HPAEC-PAD measurement of Ino and Gol. HPAEC-PAD chromatogram of WSCs and sugar alcohols extracted from (A) WT seeds and 100 µM Ino (C) and Gol (B) as reference compounds.

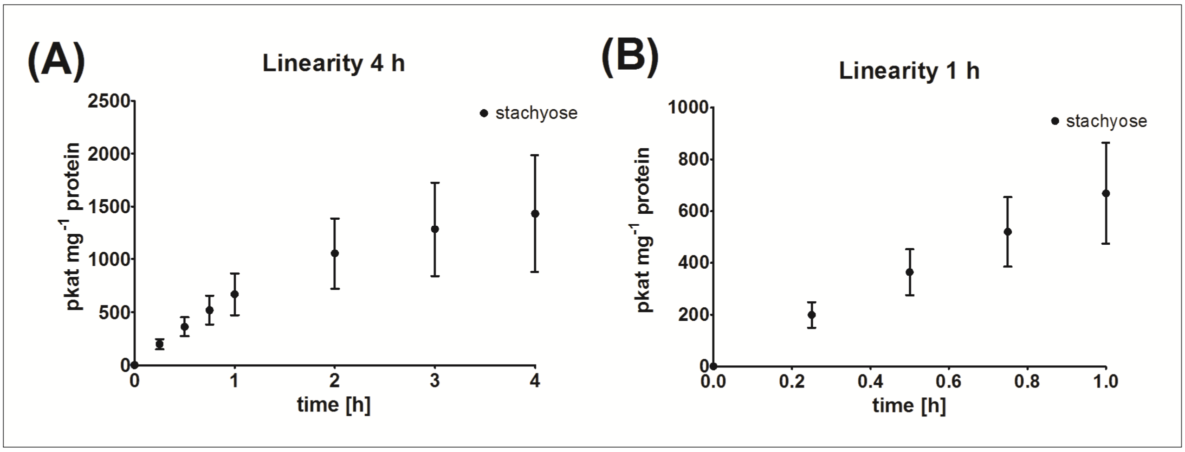

Supplementary Figure S5 Linearity of Sta product formation in HPAEC-PAD enzyme assay.

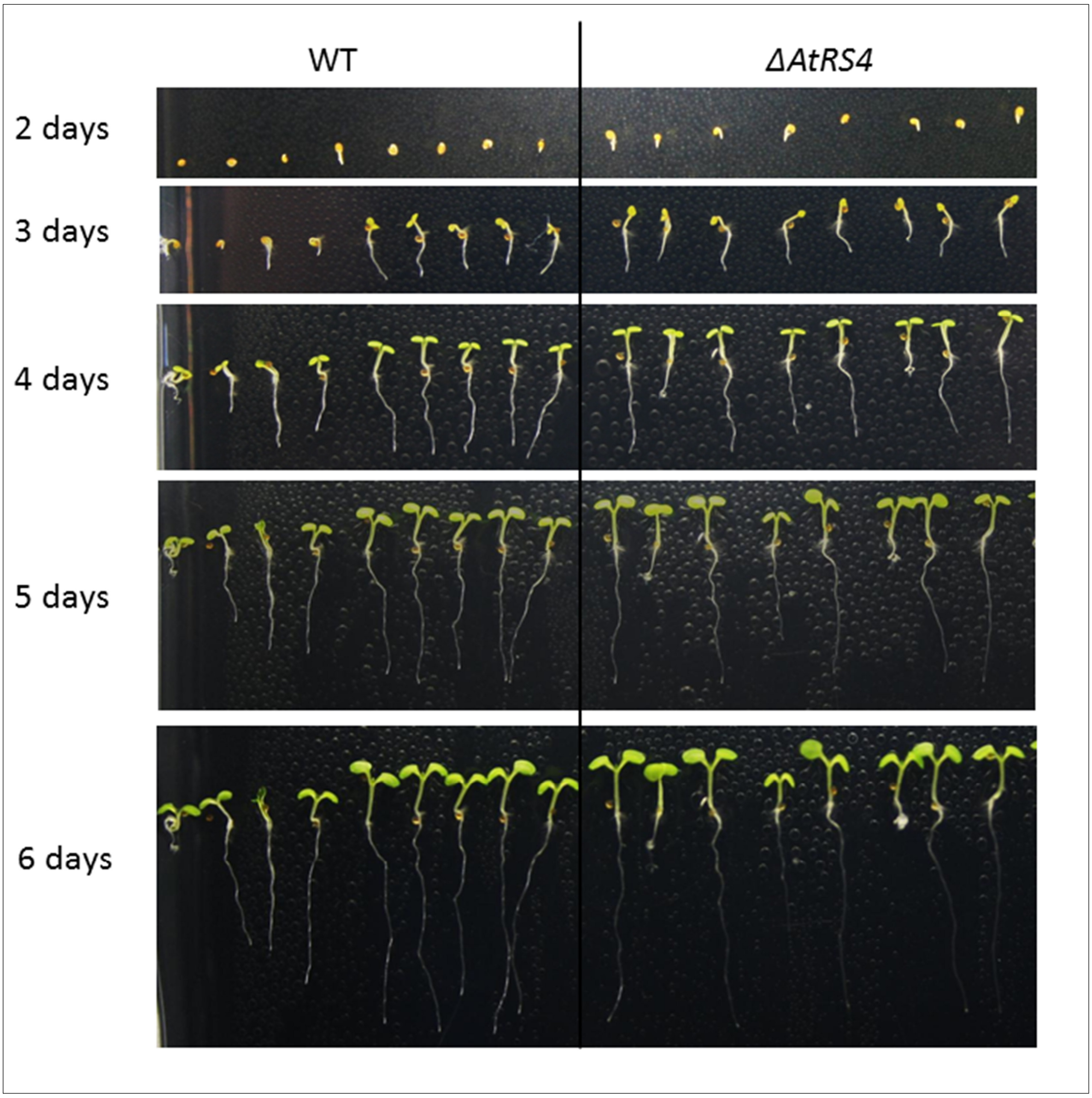

Supplementary Figure S6 Germination of WT and *ΔAtRS4* mutant seeds on 0.5 x MS agar plates.

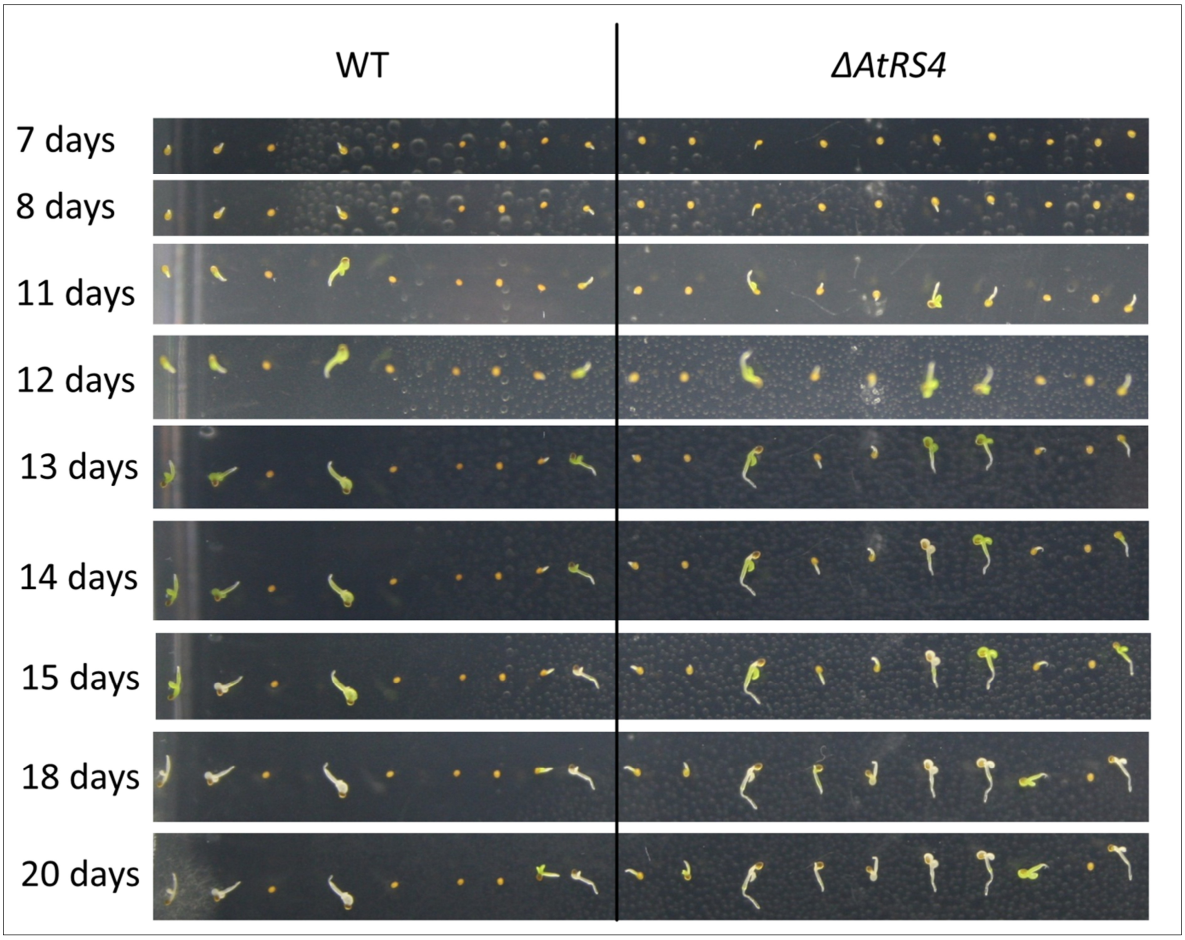

Supplementary Figure S7 Germination of WT and *ΔAtRS4* mutant seeds on 0.5 x MS agar plates supplemented with 200 mM NaCl.

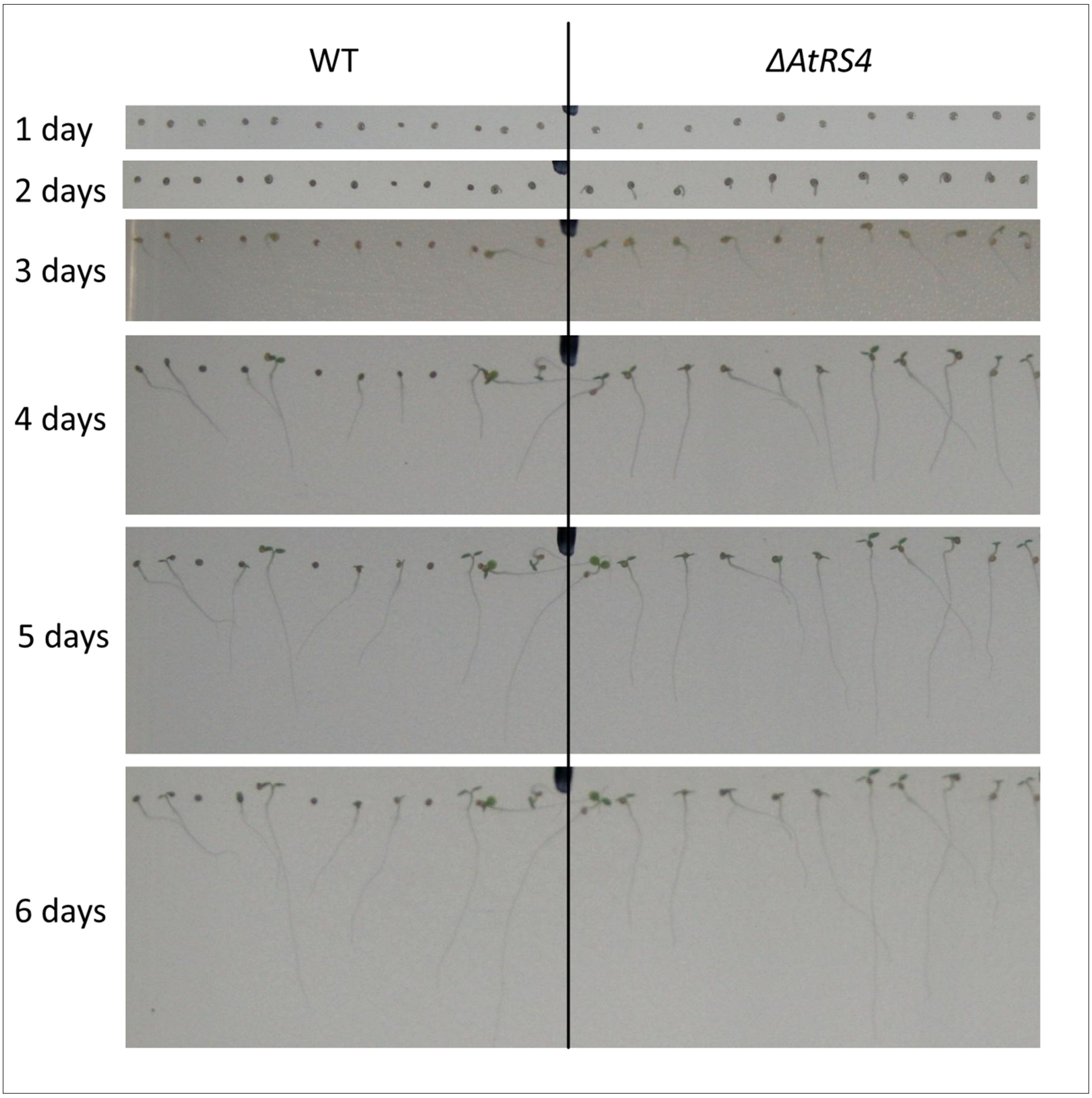

Supplementary Figure S8 Germination of WT and *ΔAtRS4* mutant seeds on 0.5 % agar plates.

1.2 Supplementary Tables

Supplementary Table S1 Primer sequences.

Primer	Sequence
wtAtRS4_fwd	5′-AAGGAGGGTTTCTCGGATTC-3’
wtAtRS4_rev	5′-TCTCAGCGTCTTTGTCTAGC-3’
SALK left border	5′-ACTCAACCCTATCTCGGGCTATTC-3’
AtRS4_fwd	5’-CCGAGCTCATGGCTCCACTTCAC-3’
AtRS4_rev	5’-TTCCCGGGTTAAAAGGTGAAAGACAGATG-3’
qAtRS4_fwd	5’-GCTGGGATTGGTCTTGTTCATCC-3’
qAtRS4_rev	5’-TTCTGCTAGTGACTCCAAGGTTTG-3’
